# Intermolecular Aggregation‐Induced Delayed Fluorescence Scintillators for Ultrahigh‐Resolution X‐Ray Imaging

**DOI:** 10.1002/advs.75096

**Published:** 2026-04-03

**Authors:** Jie Yuan, Ying Liu, Botao Zheng, Xu Zhao, Yongrong Wang, Peng Zhang, Yifei Liu, Jingxia Zheng, Ping Li, Jianwei Li, Shen Xu, Runfeng Chen, Ye Tao

**Affiliations:** ^1^ State Key Laboratory of Flexible Electronics (LoFE) & Institute of Advanced Materials (IAM) Nanjing University of Posts & Telecommunications Nanjing China; ^2^ Nanjing University of Industry Technology Nanjing China; ^3^ School of Computer Science and Technology Shandong University Qingdao China

**Keywords:** aggregation‐induced delayed fluorescence, high spatial resolution, organic X‐ray scintillators, thin‐film, X‐ray imaging

## Abstract

Organic X‐ray scintillators (OXSTs) with excellent optoelectronic properties and facile processability are highly attractive for next‐generation radiation detection and medical imaging. However, achieving efficient solid‐state emission with high exciton utilization efficiency remains a formidable challenge because aggregation‐caused quenching fundamentally suppresses radiative processes. Here, we introduce an intermolecular aggregation‐induced delayed fluorescence (AIDF) strategy that combines aggregation‐induced emission and thermally activated DF features. The tailored emitter exhibits efficient AIDF with sub‐microsecond delayed lifetimes via through‐space charge transfer, enabling effective harvesting of singlet and triplet excitons. When blended into a polysulfone host, the composite films deliver bright and stable radioluminescence across a wide concentration range, showing exceptional resistance to concentration quenching under X‐ray excitation. Benefiting from large Stokes shifts, rapid reverse intersystem crossing, and efficient radiative emission, the resulting scintillator achieves an ultralow detection limit of 0.255 µGy s^−1^ and a high spatial resolution of 20.0 lp mm^−1^, outperforming most organic counterparts and even some inorganic ones. The flexible, uniform, and transparent scintillation screens also exhibit excellent photostability for high‐quality X‐ray imaging. This work establishes a practical molecular design paradigm to circumvent aggregation‐caused quenching and highlights the unique advantages of intermolecular AIDF in enabling efficient exciton utilization, paving the way for high‐resolution thin‐film scintillators.

## Graphical abstract

An intermolecular AIDF strategy enables an organic X‐ray scintillator (OXST) that resists concentration quenching and efficiently utilizes singlet and triplet excitons. The AIDF emitter, embedded in a polysulfone matrix, yields bright, flexible, and transparent scintillation screens with ultralow detection limits and high spatial resolution, offering a practical route toward high‐performance OXSTs.

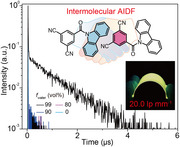

## 1 Introduction

Scintillators are essential functional materials in X‐ray imaging systems, enabling applications in medical diagnostics, high‐energy physics, industrial nondestructive testing, and space exploration [1, 2, 3]. Recently, flexible thin‐film scintillators have emerged as transformative alternatives to conventional rigid single‐crystal counterparts. Their advantages stem from inherent flexibility, enabling conformal contact with nonplanar surfaces for high‐fidelity imaging of irregular objects, and from adaptability to nonuniform X‐ray irradiation, thereby minimizing vignetting artifacts in large‐area imaging [4, 5]. Current research efforts are focused on metal halide perovskites and metal nanoclusters, owing to their strong X‐ray absorption, high photoluminescence quantum yields, and compatibility with solution‐based thin‐film fabrication on flexible substrates [5, 6, 7, 8]. These features highlight their potential in low‐dose X‐ray imaging and next‐generation or wearable detectors. Beyond these inorganic platforms, organic X‐ray scintillators (OXSTs) with excellent optoelectronic properties and versatile processability are emerging as transformative alternatives to conventional inorganic counterparts, offering unique advantages that substantially expand the applicability of X‐ray imaging technologies [9, 10, 11]. However, the use of OXSTs in high‐resolution imaging remains severely limited by intrinsic weak X‐ray absorption ability, inefficient exciton utilization, and aggregation‐caused quenching (ACQ) in the solid state.

Aggregation‐induced emission (AIE), pioneered by Tang and coworkers, features a core mechanism that transforms molecular packing from a luminescence‐quenching factor into an enhancing one, providing a highly efficient strategy to overcome ACQ [12, 13, 14]. AIE luminogens retain strong emissive properties even in dense solid states or at high concentrations, endowing them with broad application prospects in solid‐state radiation detection and high‐loading scintillation systems [15, 16, 17]. However, despite the significant improvement in radiative efficiency of AIE in aggregated states, it inherently fails to address the critical issue of low triplet exciton utilization efficiency that restricts the performance of numerous organic luminogens under high‐energy X‐ray excitation [18].

Aggregation‐induced delayed fluorescence (AIDF) provides a more advanced exciton‐regulation mechanism by synergistically integrating the solid‐state tolerance of AIE with the triplet‐harvesting capability of thermally activated delayed fluorescence (TADF) [19, 20, 21]. Unlike conventional TADF materials, which commonly suffer from concentration sensitivity or ACQ effects, AIDF relies on aggregation‐induced intermolecular interactions to not only stabilize the molecular configuration of luminogens and suppress nonradiative decay pathways but also significantly accelerate the reverse intersystem crossing (RISC) rate, thereby maintaining efficient luminescence over a wide doping concentration range while simultaneously enabling triplet exciton utilization [22, 23].

In this work, inspired by high exciton utilization efficiency and concentration quenching resistance in dense solid matrices, an intermolecular AIDF scintillator was designed and synthesized by simply incorporating carbazole chromophore into isophthalonitrile [24]. The resulting scintillator demonstrates efficient exciton utilization, a rapid RISC rate of 3.91 × 10^6^ s^−1^, large Stokes shifts of 248 nm, and stable radioluminescence (RL) emission across broad concentration ranges. Both experimental and theoretical analyses indicate that intermolecular AIDF reduces singlet‐triplet energy gaps (Δ*E*_ST_) via through‐space charge transfer (TSCT), accelerates RISC, and suppresses nonradiative decay [20, 25]. Moreover, homogeneous dispersion of AIDF emitters in polymer matrices enables uniform and flexible large‐area films with minimized light scattering and weak self‐absorption. Flexible scintillator screens exhibit ultralow detection limits of 0.255 µGy s^−1^, and a high spatial resolution of 20.0 lp mm^−1^, surpassing most reported organic and even some inorganic scintillators [3, 26, 27, 28]. These results underscore the enormous potential of AIDF luminogens for developing high‐performance solution‐processable and high‐resolution OXSTs technologies.

## 2 Results and Discussion

### 2.1 Material Synthesis and Characterization

**FIGURE 1:**
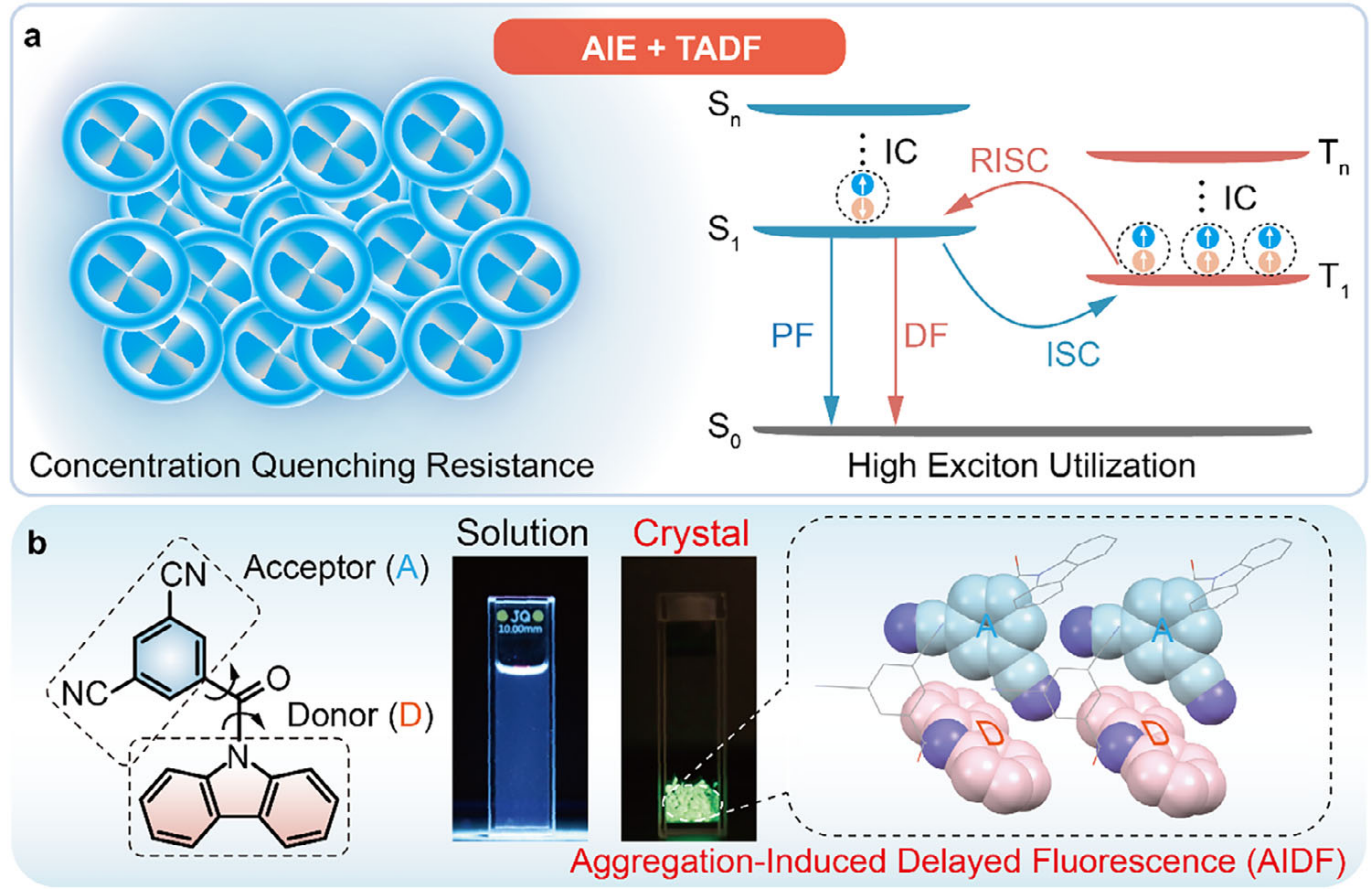
(a) Schematic illustration showing the incorporation of aggregation‐induced emission (AIE) characteristics into thermally activated delayed fluorescence (TADF) systems. (b) The molecular structure, luminescence photographs, and 3D crystal structure illustrating the spatial arrangement of donor and acceptor moieties of the aggregation‐induced delayed fluorescence (AIDF) molecule.

In theory, incorporating AIE characteristics into TADF systems can effectively suppress ACQ and increase exciton utilization efficiency, yet achieving both AIE and TADF within a single molecular system remains challenging (Figure 1a) [29, 30]. Inspired by exciplex‐type TADF, which arises from the formation of an excited‐state complex via intermolecular electron transfer upon blending electron donor (D) and electron acceptor (A) materials, we developed an intermolecular AIDF molecule in which molecules adopt a twisted donor–acceptor (D–A) framework bridged by carbonyl linkers [31, 32, 33]. This architecture suppresses intramolecular charge transfer (ICT) interactions at the isolated molecule level, while in the aggregated state, electrostatic interactions between neighboring donor and acceptor units bring molecules into close proximity, forming TSCT states (Figure 1b) [34, 35]. TSCT enables efficient charge transfer (CT) via spatial orbital overlap, minimizing exchange energy and narrowing Δ*E*_ST_, thereby facilitating efficient RISC and robust TADF in the solid state.

To validate this concept, an intermolecular AIDF emitter, 5‐(9H‐carbazole‐9‐carbonyl)isophthalonitrile (**DCCz**), was synthesized using isophthalonitrile as the electron acceptor and carbazole as the electron donor, bridged through a carbonyl linker. **DCCz** was obtained in high yield through a straightforward multi‐step reaction (Scheme S1). Detailed synthetic procedures, molecular structure characterization, elemental analysis, thermal stability assessment, single‐crystal X‐ray diffraction, and electrochemical properties are provided in the Supporting Information (Figures S1–S10 and Table S1).

### 2.2 Photophysical Property Investigation

**FIGURE 2:**
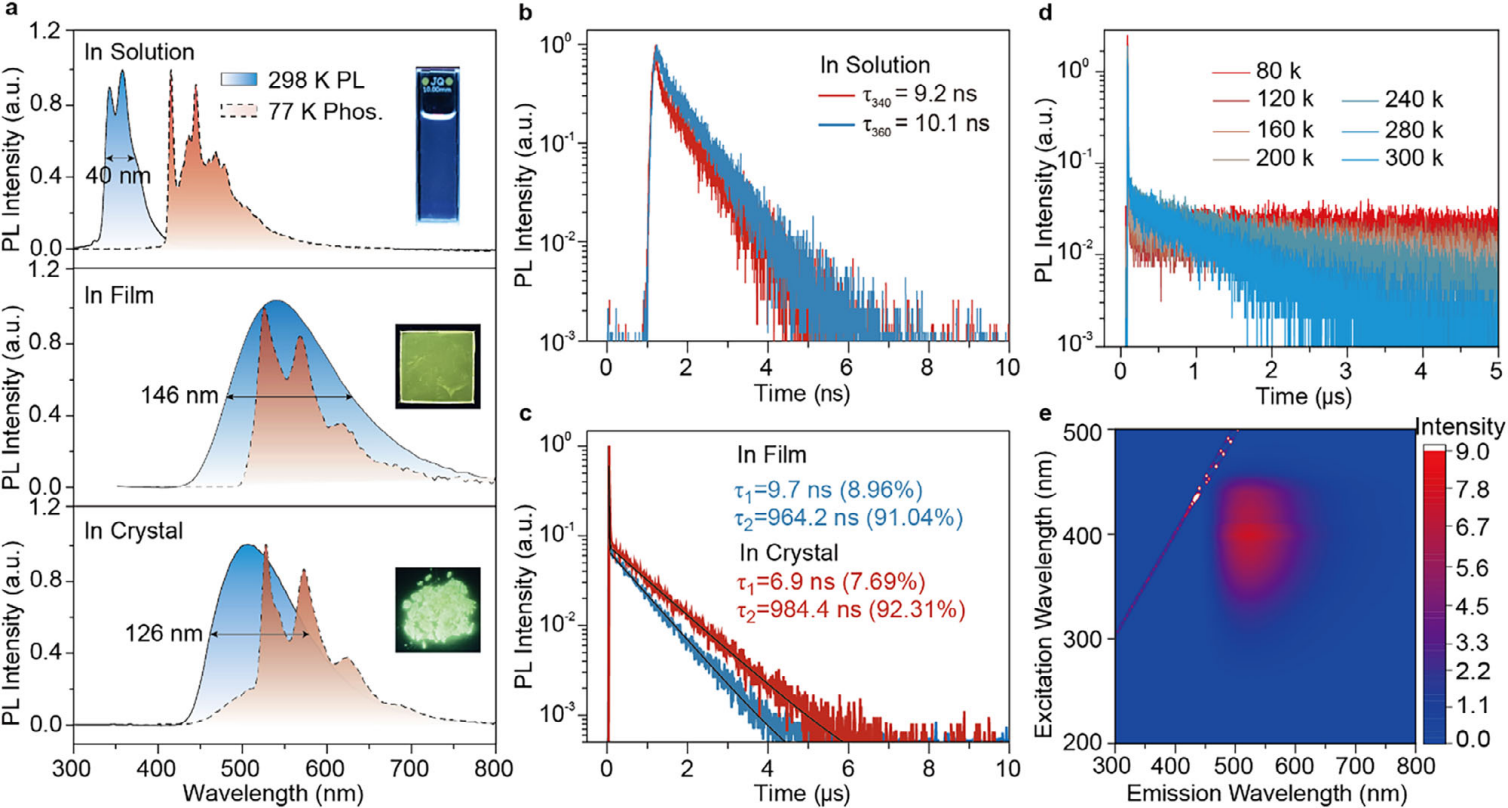
(a–c) Room‐temperature fluorescence, low‐temperature delayed PL spectra (delay time: 10 ms) (a) and lifetime decay profiles (b, c) of **DCCz** in dichloromethane (DCM) solution(∼10^−5^ m), doped film (in polysulfone), and crystal excited at 295 nm. (d) Temperature‐dependent transient PL decay curves of **DCCz** in a crystal. (e) Excitation‐emission mapping of the doped film (in polysulfone) at room‐temperature.

**DCCz** exhibits UV–vis absorption spectra that closely resemble those of carbazole in both dichloromethane (DCM) dilute solution and pure film (Figure S11) [36]. In DCM dilute, characteristic fluorescence bands at 300–400 nm with distinct vibronic structures from the carbazole moiety were observed, indicating negligible ICT effects at the isolated molecule level [37, 38, 39]. Remarkably, in the doped film or in the crystalline state, the monomolecular carbazole emission vanished completely, and only bright yellow emission was observed (Figure 2a). Compared with the dilute solution, these emission ranging from 425 to 750 nm was markedly redshifted and broadened, characteristic of CT state emission. This effect was also pronounced in low‐polarity solvents with limited solubility, such as *n*‐hexane and toluene, suggesting the formation of a new excited state intramolecular charge transfer (Figure S12) [40]. Besides, the slight blueshift for the crystal relative to the 10 wt.% doped film was observed, which can be mainly attributed to the more rigid and ordered molecular packing in the single crystal. Such a highly ordered environment restricts vibrational relaxation and thus reduces the Stokes shift. In contrast, the doped film exhibits looser molecular packing and greater conformational freedom, resulting in a redshifted emission. Concentration‐dependent photoluminescence measurements in *n*‐hexane, toluene, DCM, tetrahydrofuran, and acetonitrile revealed that, as the concentration increased from 10^−7^ to 10^−4^ m, a broad CT emission band emerged in the 450–700 nm region in addition to the carbazole emission (Figure S13). Thus, the excited states formed via TSCT between the carbazole (donor) moieties of one molecule and the isophthalonitrile (acceptor) moieties of adjacent molecules in the aggregated state, rather than intramolecular charge transfer within an isolated single molecule. The AIDF feature was also confirmed by the time‐resolved photoluminescence spectra. Compared to the typical fluorescence with nanosecond lifetimes (< 10 ns) in dilute solution, whereas in doped film and crystal, a delayed fluorescence component with lifetimes approaching 1 µs was observed, accounting for over 90% of the total emission (Figure 2b,c) [41, 42, 43].

To elucidate the origin of the delayed emission, the photoluminescence behavior of **DCCz** was examined in THF/water mixtures. With increasing water fraction, the monomolecular emission at 340–360 nm gradually decreased, while a new broad‐band emission in the 450–700 nm region emerged and intensified, indicative of aggregate formation and characteristic AIE behavior (Figure S14a) [44]. Fluorescence lifetime measurements at 520 nm revealed progressively prolonged lifetimes with biexponential decay profiles, suggesting an enhanced contribution from delayed fluorescence upon aggregation, which is consistent with AIDF characteristics (Figure S14b) [40]. Furthermore, delayed PL spectra recorded in solution, doped film, and crystalline states yielded Δ*E*_ST_ of 1.22, 0.01, and 0.09 eV, respectively (Figure 2a). The small Δ*E*_ST_ values in the doped film and crystalline state fulfill the energy requirement for the efficient RISC process. Temperature‐dependent lifetime measurements of **DCCz** crystals (80–300 K) further demonstrated biexponential decay dynamics. With the temperature increases, its relative contribution to the total emission increases due to the thermally activated RISC, corroborating TADF behavior (Figure 2d and Table S2) [45, 46]. Therefore, these results provide solid evidence that **DCCz** exhibits intermolecular AIDF in both films and crystalline states.

Furthermore, the RISC rate constant (*k*_RISC_) of **DCCz** in the crystalline state was calculated, with the detailed data provided in Table S3. Notably, no detectable RISC process was observed in solution, whereas in the crystalline state, **DCCz** exhibited a rapid RISC process with a rate constant of 3.91 × 10^6^ s^−1^. The rate is relatively fast within delayed fluorescence molecular systems, which can be attributed to the synergistic enhancement arising from AIE effects for the formation of TSCT that largely reduces the overlap of frontier molecular orbital [37, 47]. Steady‐state excitation‐emission mapping indicated that the **DCCz**‐doped films exhibit a stable emission band spanning 460–620 nm when excited in the 325–450 nm range (Figure 2e). This emission band shows excellent spectral overlap with the response window of SiPM detectors (Figure S15). Moreover, it exhibits minimal overlap with the absorption bands of **DCCz** in doped films, showing large Stokes shifts of 248 nm for effectively suppressing self‐absorption.

### 2.3 X‐ray Scintillation Properties

**FIGURE 3:**
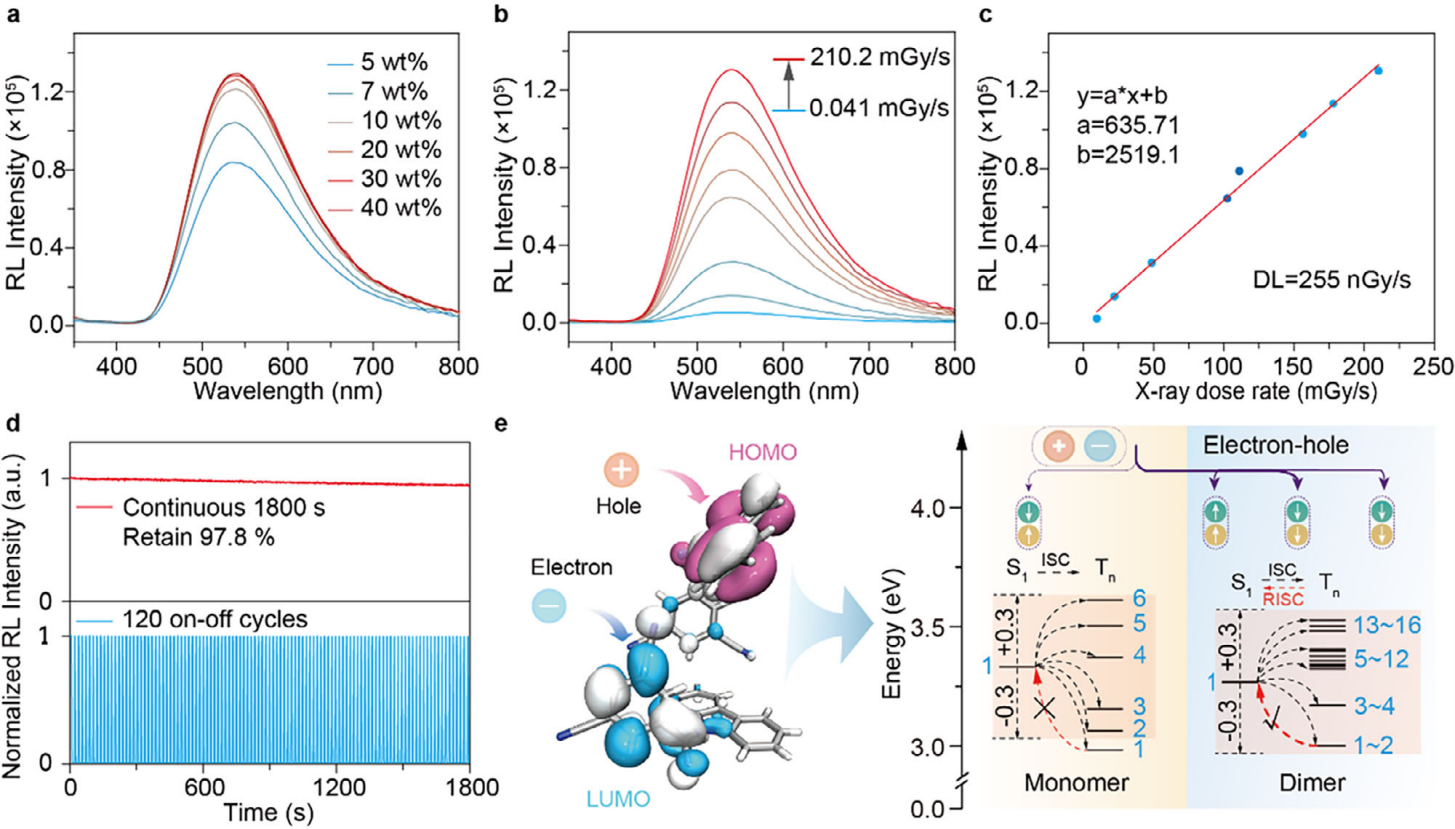
(a) RL spectra of **DCCz**/PSF doped films at different doping concentrations. (b) Dosage‐dependence and (c) dose rate dependence of RL intensity in the range of 0.041 to 210.2 mGy s^−1^ of 10 wt% **DCCz**/PSF film. (d) The X‐ray photostability for 10 wt% **DCCz**/PSF films under continuous irradiation (top) and repeated on‐off cycles (bottom) at a dose rate of 210.0 mGy s^−1^ under ambient conditions. (e) Schematic illustration of the radiative emission mechanism in AIDF luminogens, the reduced singlet‐triplet energy gap (Δ*E*_ST_) facilitates the conversion of triplet excitons to the lowest singlet state (S_1_) via reverse intersystem crossing (RISC) in dimeric aggregates, complemented by TD‐DFT‐calculated energy levels of the lowest singlet (S1) and triplet (*T_n_*, *n* = 1, 2,…) states within the range of 0.3 eV. [Correction added on 6 April 2026 after first online publication: Figure 3 is updated.]

The as‐prepared AIDF materials exhibit excellent photoluminescent properties, highlighting their promise for X‐ray scintillation applications. To evaluate their RL performance, **DCCz**‐doped polysulfone (PSF) films with varying doping concentrations were fabricated (Figure S16). PSF was selected as the host matrix owing to its high optical transparency in the UV–visible range, which facilitates efficient light extraction, as well as its high glass transition temperature and mechanical flexibility, which ensure stability under high‐energy irradiation [48, 49]. As illustrated in Figure S17, the absence of heavy atoms in **DCCz** and PSF results in negligible absorption coefficients (**DCCz**, *Z*_max_ = 8, *K*_α_ = 0.5 keV; PSF, *Z*_max_ = 16, *K*_α_ = 2.3 keV). The RL intensities increased progressively with the doping concentration in the range of 3 to 40 wt.%, stabilizing above 30 wt.% (Figure 3a). This trend can be attributed to the enhanced radiative transition emission arising from the increased ratio of luminophores. However, phase separation at higher loadings (above 30 wt.%) not only reduces film transparency but also attenuates the transfer of the excitons from the host to the guest (Figure S18). Notably, the RL intensity remains at its maximum even at a high doping concentration of 40 wt.%, with no ACQ phenomenon observed. Importantly, across the entire doping range of 3–40 wt.%, the emission peak was consistently maintained at about 540 nm, confirming excellent spectral stability [50, 51].

Moreover, with increasing X‐ray dose rates, the RL intensities of the films exhibited a linear enhancement (Figure 3b). The detection limit (DL) of the 10 wt.% **DCCz**/PSF film was calculated to be 0.255 µGy s^−1^, which is far below the standard dose used in medical X‐ray imaging (5.5 µGy s^−1^), highlighting its excellent sensitivity (Figure 3c) [50]. In addition, the **DCCz**/PSF film displayed remarkable radiation stability, its emission intensity remained nearly unchanged after 120 on/off excitation cycles, and only a 2.2% RL loss was observed after continuous X‐ray irradiation for 30 min (Figure 3d). This remarkable robustness can be attributed to the spatial separation of radiation‐generated secondary electrons and holes into different molecules, thereby preventing charge accumulation and degradation of individual AIDF molecules (Figure 3e).

To gain deeper insight into the structure‐property relationship, quantum chemical calculations were carried out. In the **DCCz** dimer, the highest occupied molecular orbital (HOMO) is predominantly localized on the carbazole unit of one molecule, while the lowest unoccupied molecular orbital (LUMO) is mainly distributed over the isophthalonitrile unit of the neighboring molecule in the dimer (Figure 3e). This spatial separation of frontier orbitals leads to a complete decoupling of the HOMO and LUMO, thereby reducing the Δ*E*_ST_, lowering the exchange energy (from 0.122 to 0.006 eV), and promoting efficient RISC [32]. Theoretical results are consistent with the experimentally determined Δ*E*_ST_ (< 0.1 eV), further validating the feasibility of intermolecular AIDF (Table S4).

Based on these findings, we propose the following RL mechanism for AIDF emitters. Upon X‐ray irradiation, the PSF host efficiently absorbs X‐rays and generates high‐energy electrons through the photoelectric effect and Compton scattering. These thermalized electrons subsequently interact with molecular atoms, producing abundant low‐energy electrons and holes that recombine in a 1:3 ratio to yield singlet and triplet excitons. In AIDF systems, the spatially separated molecular configuration significantly reduces Δ*E*_ST_, thereby enabling rapid RISC from triplet to singlet states and ensuring exciton utilization efficiency (Figure 3e).

### 2.4 Imaging Applications

**FIGURE 4:**
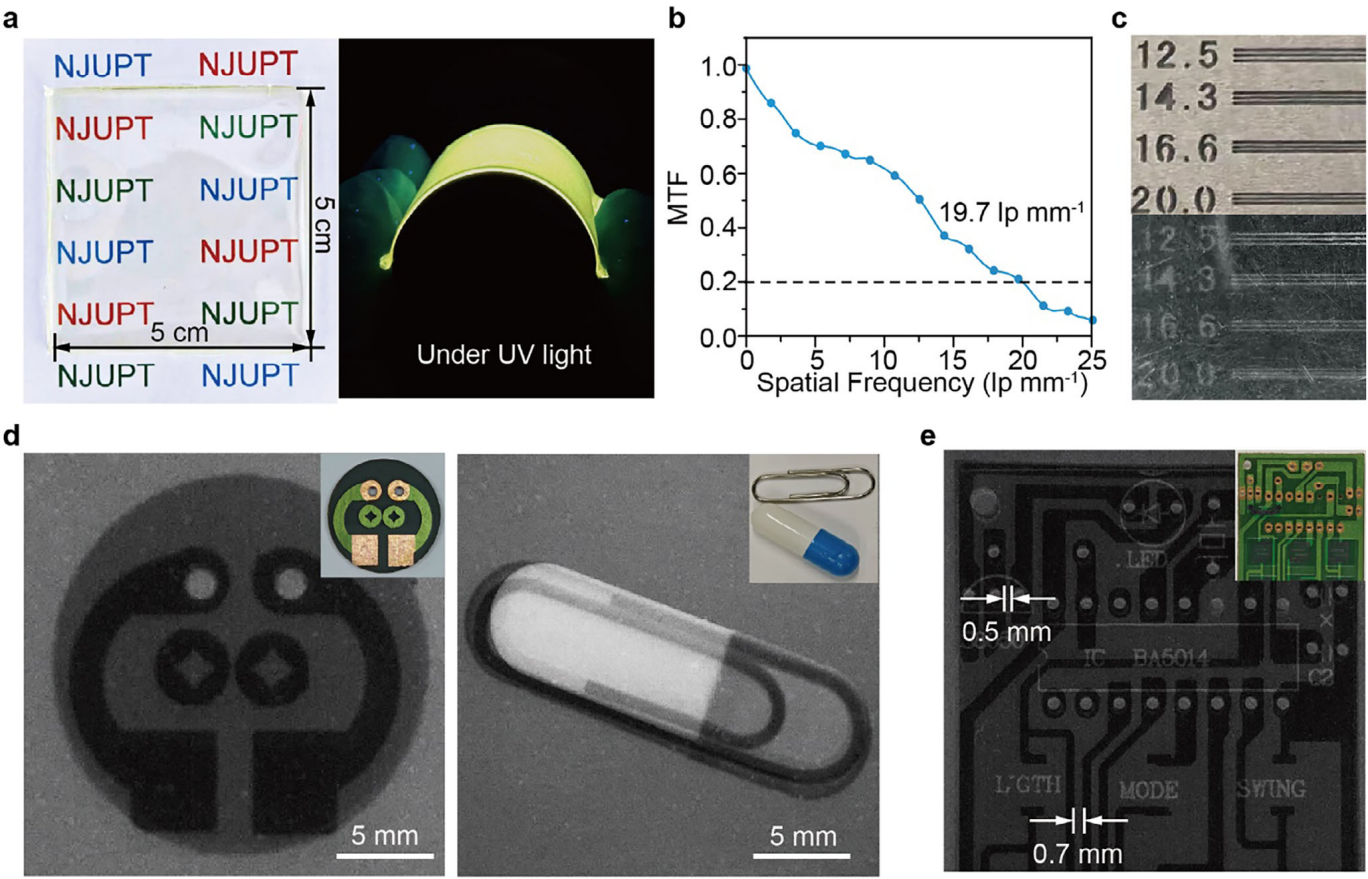
(a) Photograph of the **DCCz**/PSF scintillation screen under bright‐field illumination (left) and under UV light (right). (b) Modulation transfer function (MTF) derived from the X‐ray slanted‐edge image. (c) X‐ray imaging of the standard resolution test plate: bright‐field (top) and corresponding X‐ray (bottom) images of a partial region (12.5–20 lp mm^−1^). (d, e) X‐ray excited images of printed circuit board, ackaged paper clips (d), and an electronic chip (e), with insets showing the corresponding bright‐field photographs.

The imaging performance of the AIDF scintillation screen was systematically evaluated. Owing to its outstanding X‐ray scintillation properties, the 10 wt.% doped **DCCz**/PSF film (5 × 5 cm) was produced as the scintillator screen (Figure S16). The doped films exhibited excellent uniformity, flexibility, and optical transparency (Figure 4a). The spatial resolution of the scintillation screen was quantitatively assessed using the modulation transfer function (MTF). A standard X‐ray slanted‐edge image revealed a resolution of 19.7 lp mm^−1^ at an MTF value of 0.2, obtained via edge spread function analysis (Figure 4b) [11]. Consistently, X‐ray imaging of a standard resolution test chart showed that both the bright‐field photograph and the corresponding X‐ray image displayed clearly resolvable line patterns with a spacing of 20.0 lp mm^−1^ using a standard line‐pair test pattern under real imaging conditions, surpassing most reported organic and even inorganic scintillators (Figure 4c and Table S5). In addition, a series of practical imaging tests was carried out to validate the applicability of the flexible thin‐film scintillator screen under realistic conditions. Under X‐ray irradiation, a commercial digital camera successfully captured fine structural details such as printed circuit board and ackaged paper clips in opaque capsules (Figure 4d). When an opaque electronic chip with a complex internal architecture was placed between the X‐ray source and the scintillation screen, the flexible thin‐film scintillator screen produced X‐ray images with distinct brightness contrasts, clearly delineating otherwise hidden internal structures (Figure 4e). These results highlight the significant potential of AIDF‐based scintillator screens for high‐resolution X‐ray imaging and nondestructive testing.

## 3 Conclusion

In conclusion, we have demonstrated a viable molecular engineering strategy toward high‐performance OXSTs through rational intermolecular AIDF design by synergistically integrating AIE and TADF. Benefiting from TSCT, the tailored emitters exhibit sub‐microsecond AIDF characteristics, enabling efficient singlet/triplet harvesting and effectively circumventing ACQ. When embedded into the PSF matrix, the resulting composite films deliver bright and stable X‐ray luminescence with remarkable resistance to concentration quenching. In particular, the OXSTs feature large Stokes shifts, rapid RISC and high emission efficiency, achieving an ultralow detection limit of 0.255 µGy s^−1^ and high spatial resolution up to 20.0 lp mm^−1^. Moreover, the flexible, uniform, and transparent scintillating screens exhibit excellent photostability, ensuring reliable imaging performance. This study not only underscores the potential of intermolecular AIDF in enhancing exciton utilization and suppressing non‐radiative loss but also provides an effective approach to mitigate ACQ, opening avenues toward flexible thin‐film scintillators for applications in commercial radiography.

## Funding

This work was supported in part by the National Natural Science Foundation of China (22322106 and 62288102), Basic Research Program of Jiangsu (BK20243057), Natural Science Foundation of Jiangsu Province (BK20240640), the Jiangsu Specially‐Appointed Professor Plan, U35 Strong Foundation Program of Nanjing, Natural Science Research Start‐up Foundation of Recruiting Talents of Nanjing University of Posts and Telecommunications (NY224003), the Hua Li Talents Program of Nanjing University of Posts and Telecommunications and the Open Research Fund of State Key Laboratory of Organic Electronics and Information Displays.

## Conflicts of Interest

The authors declare no conflicts of interest.

## Supporting information




**Supporting File**: advs75096‐sup‐0001‐SuppMat.docx.

## Data Availability

The data that support the findings of this study are available in the supplementary material of this article.
