# Supplementary material for: Intermolecular Aggregation‐Induced Delayed Fluorescence Scintillators for Ultrahigh‐Resolution X‐Ray Imaging
Source: Adv Sci (Weinh). 2026 Apr 3;13(34):e75096. doi: 10.1002/advs.75096 (PMC13285166; doi:10.1002/advs.75096)
Supplement: Supplementary file 1 — Supporting File: advs75096‐sup‐0001‐SuppMat.docx. [file ADVS-13-e75096-s001.docx]

Supporting Information

# Intermolecular Aggregation-Induced Delayed Fluorescence Scintillators for Ultrahigh-Resolution X-Ray Imaging

*Jie Yuan^†^, Ying Liu^†*^,* *Botao Zheng, Xu Zhao, Yongrong Wang,* *Peng Zhang,* *Yifei Liu, Jingxia Zheng, Ping Li, Jianwei Li, Shen Xu^*^, Runfeng Chen and Ye Tao^*^*

1State Key Laboratory of Flexible Electronics (LoFE) & Institute of Advanced Materials (IAM), Nanjing University of Posts & Telecommunications, 9 Wenyuan Road, Nanjing 210023, China.

2Nanjing University of Industry Technology, 1 Yangshan North Road, Nanjing 210023, China.

3School of Computer Science and Technology, Shandong University, 72 Binhai Road, Qingdao 266237, China.

*Corresponding Author(s): Ying Liu: liuying01@sdu.edu.cn; Shen Xu: iamsxu@njupt.edu.cn; Ye Tao: iamytao@njupt.edu.cn.

^†^ These authors contributed equally to this work.

**Content**

[1. Synthesis and Characterization 2](#_Toc212799151)

[2. Thermal Property 9](#_Toc212799152)

[3. Single Crystals Analysis 10](#_Toc212799153)

[4. Electrochemical Property 11](#_Toc212799154)

[5. Optical Properties 12](#_Toc212799155)

[6. Theoretical Calculations 16](#_Toc212799156)

[7. Fabrication of Scintillation Screen 16](#_Toc212799157)

# Synthesis and Characterization

Unless otherwise noted, chemicals and solvents purchased from Energy Chemical are of analytical grade, and were used without further purification. Manipulations involving air-sensitive reagents were performed in an atmosphere of dry argon. ^1^H and ^13^C-nuclear magnetic resonance (NMR) spectra were recorded on a Bruker Ultra Shield Plus 400 MHz instrument with CDCl_3_ or DMSO-*d*_6_ as the solvent and tetramethylsilane (TMS) as the internal standard. Elemental analysis was performed on an Elementar Vario MICRO elemental analyzer.^[^[^1^](#_ENREF_1)^]^


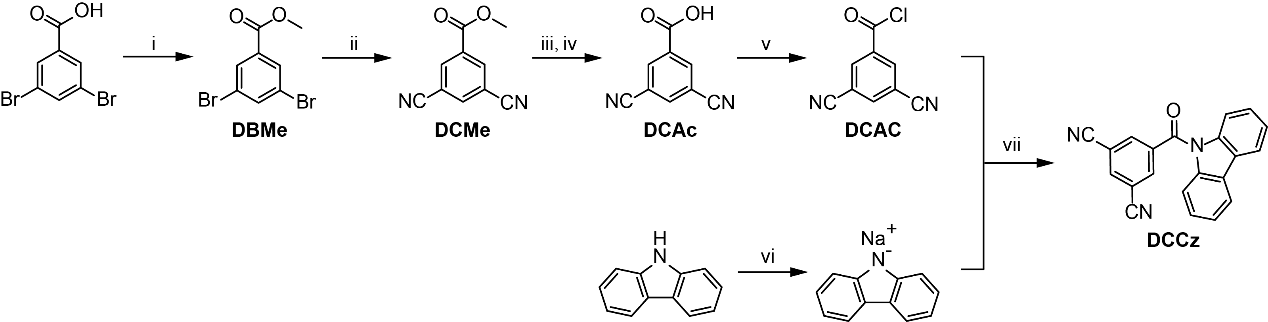


**Scheme S1**. Synthetic route for the AIDF molecule **DCCz**: (i) methanol, thionyl chloride, 70 °C, 6 h; (ii) copper(I) cyanide, N,N-dimethylformamide, 180 °C, 48 h; (iii) sodium hydroxide, rt, 4 h; (iv) hydrochloric acid, 0 °C, 4 h; (v) toluene, thionyl chloride, 70 °C, 14 h; (vi) sodium hydride, tetrahydrofuran, rt, 30 min; (vii) tetrahydrofuran, 2 h.

**Synthesis of methyl 3,5-dibromobenzoate (DBMe):** To a 250 mL round bottom flask charged with a stirring bar, thionyl chloride (3.0 mL, 40.0 mmol) was added to a stirred solution of 3,5-dibromobenzoic acid (1.40 g, 5.0 mmol) in methanol (30.0 mL), and the mixture was stirred at 70 °C for 6 h. To end the reaction, the mixture was cooled, and the reaction mixture was diluted with dichloromethane (DCM) and washed with brine for three times. The organic layers were collected and dried over MgSO_4_ and was then dried in vacuum to produce a crude powder. Then, the product was further purified by using silica gel column chromatography (petroleum ether/ DCM = 6:1) and recrystallized from DCM/hexane for several times to obtain a colorless crystal. Yield: 1.19 g (81%). ^1^H NMR (400 MHz, CDCl_3_, ppm): δ=8.12 (m, 2H), 7.87 (d, 1H), 3.95 (s, 3H); ^13^C NMR (400 MHz, CDCl_3_, ppm): δ=164.55, 138.26, 133.27, 131.38, 123.03, 52.79.

**Synthesis of methyl 3,5-dicyanobenzoate (DCMe)**: To a 100 mL round bottom flask charged with a stirring bar, a mixture of **DBMe** (2.94 g, 10.0 mmol) and cuprous cyanide (2.24 g, 25.0 mmol) in dry dimethylformamide (40.0 mL) was refluxed at 160 °C for 24 h. After cooling to room temperature, the solvent of N, N-dimethylformamide (DMF) was removed by vacuum distillation. The resulting solid was dissolved in DCM and washed with brine. The mixture was then extracted with DCM for three times. The organic phase was collected and dried over MgSO_4_. After removing the solvent, the crude product was purified by column chromatograph (petroleum ether/ DCM = 2:1) to obtain a colorless powder. Yield: 1.49 g (80%). ^1^H NMR (400 MHz, CDCl_3_, ppm): δ = 8.55 (m, 2H), 8.14 (s, 1H), 4.04 (s, 3H); ^13^C NMR (400 MHz, CDCl_3_, ppm): δ = 163.20, 138.67, 136.71, 132.95, 115.82, 114.77, 53.40.

**Synthesis of 3,5-dicyanobenzoic acid (DCAc)**: To a 500 mL round bottom flask charged with a stirring bar, a solution of **DCMe** (0.93 g, 5.0 mmol) in ethanol (50 mL) was added aqueous sodium hydroxide solution (10 mL, 1.0 M), and the mixture was stirred at room temperature for 12 h. Most of ethanol was removed under reduced pressure, and the residue was diluted with water (200 mL). The aqueous layer was washed with ethyl ether (10 mL×2), and the aqueous layer was acidified with hydrochloric acid (15 mL, 1 M) under 0 °C to pH < 6. The mixture was extracted with ethyl acetate (50 mL × 3), and the organic layer was washed with brine, dried, and evaporated to give a yellow powder. Yield: 0.86 g (100%). ^1^H NMR (400 MHz, DMSO-*d*_6_, ppm): δ = 8.69 (s, 1H), 8.57 (d, 2H); ^13^C NMR (400 MHz, DMSO-*d*_6_, ppm): δ = 164.81, 140.26, 137.42, 133.90, 117.04, 113.92.

**Synthesis of 3,5-dicyanobenzoyl chloride (DCAC)**: To a 50 mL round bottom flask charged with a stirring bar, a solution of **DCAc** (0.86 g, 5.0 mmol) in toluene (10 mL) was added thionyl chloride (0.75 mL, 10.0 mmol), and catalytic amount of dimethylformamide (0.02 mL). The mixture was stirred at 70 °C for 14 h, and concentrated under reduced pressure to give the product, which was used in the next step without further purification. Yield: 0.95 g (100%).

**Synthesis of 5-(9H-carbazole-9-carbonyl)isophthalonitrile (DCCz):** To a 50 mL round bottom flask charged with a stir bar, 60% sodium hydride (0.24 g, 6.0 mmol) was added to a solution of carbazole (3.87 g, 15 mmol) in dry tetrahydrofuran (THF) (10 mL). After the solution was stirred at room temperature for 30 min, **DCAC** (1.10 g, 5.0 mmol) in dry THF (10 mL) was added, and then the mixture was stirred at 50 °C for 2 h. After cooling, the mixture was poured into water (100 mL), and the reaction mixture was diluted with DCM and washed with brine for three times. The organic layers were collected and dried over MgSO_4_ and was then dried in vacuum to produce a crude powder. Then, the product was further purified by using silica gel column chromatography (petroleum ether/ DCM = 3:1) and recrystallized from DCM/hexane for several times to obtain a yellow-green crystal. Yield: 1.12 g (70%). ^1^H NMR (400 MHz, CDCl_3_, ppm): δ=8.21 (m, 3H), 8.06 (m, 2H), 7.46 (m, 6H); ^13^C NMR (400 MHz, CDCl_3_, ppm): δ=164.63, 138.39, 138.33, 138.01, 136.00, 127.37, 126.54, 124.70, 120.45, 115.57, 115.44, 115.14. Anal. calcd. for C_21_H_11_N_3_O: C 78.49, H 3.45, N 13.08; found: C 78.52, H 3.44, N 12.96.

**
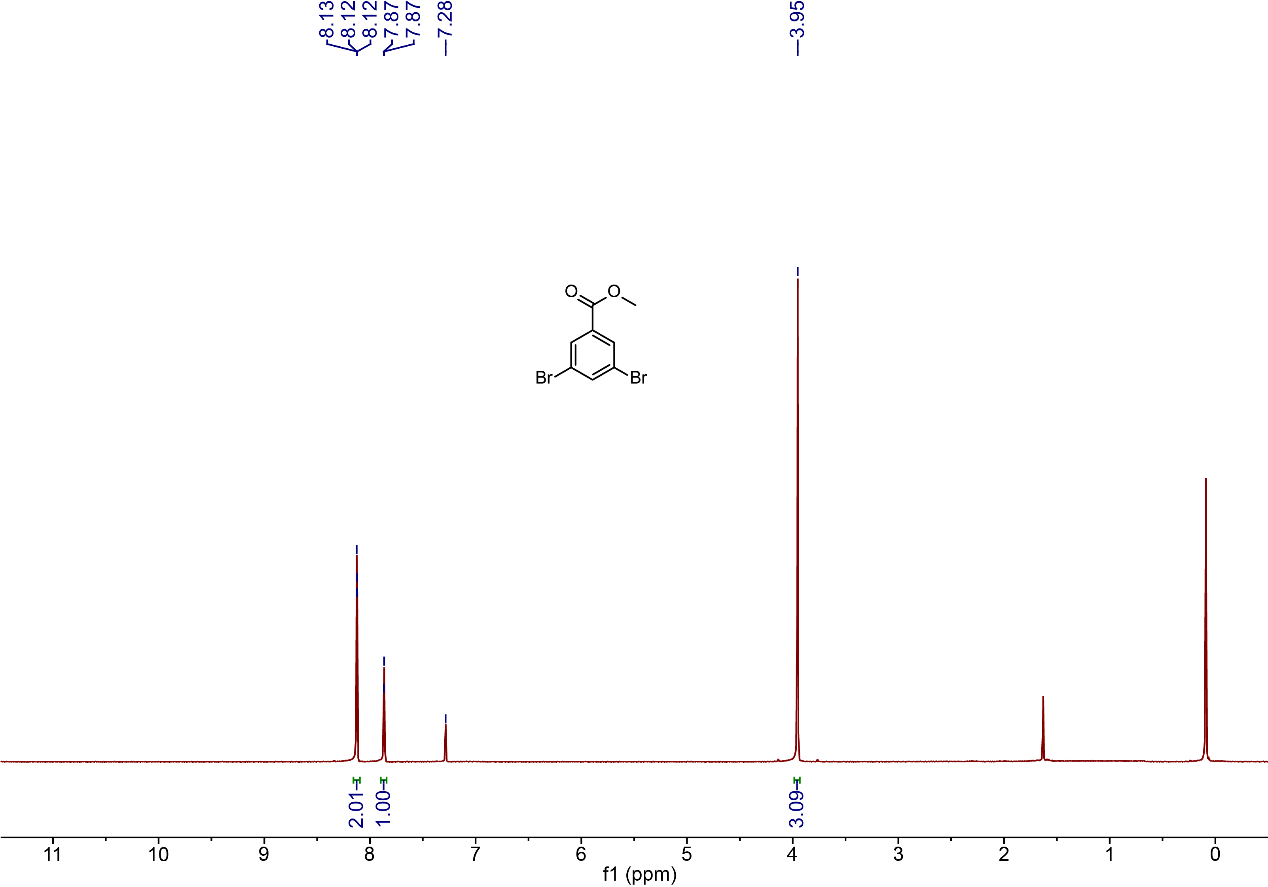
**

**Figure S1.** ^1^H NMR of **DBMe** in CDCl_3_.


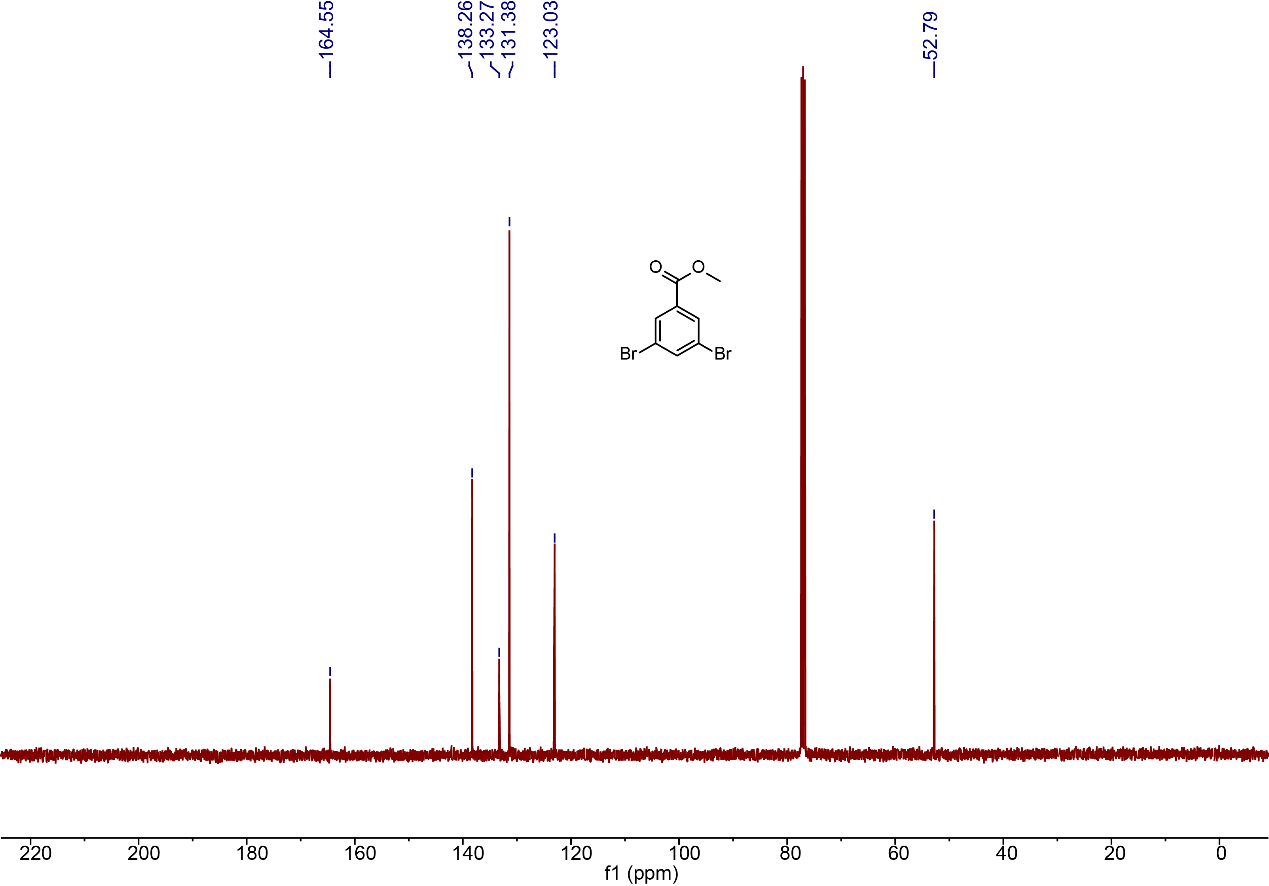


**Figure S2.** ^13^C NMR of **DBMe** in CDCl_3_.

**
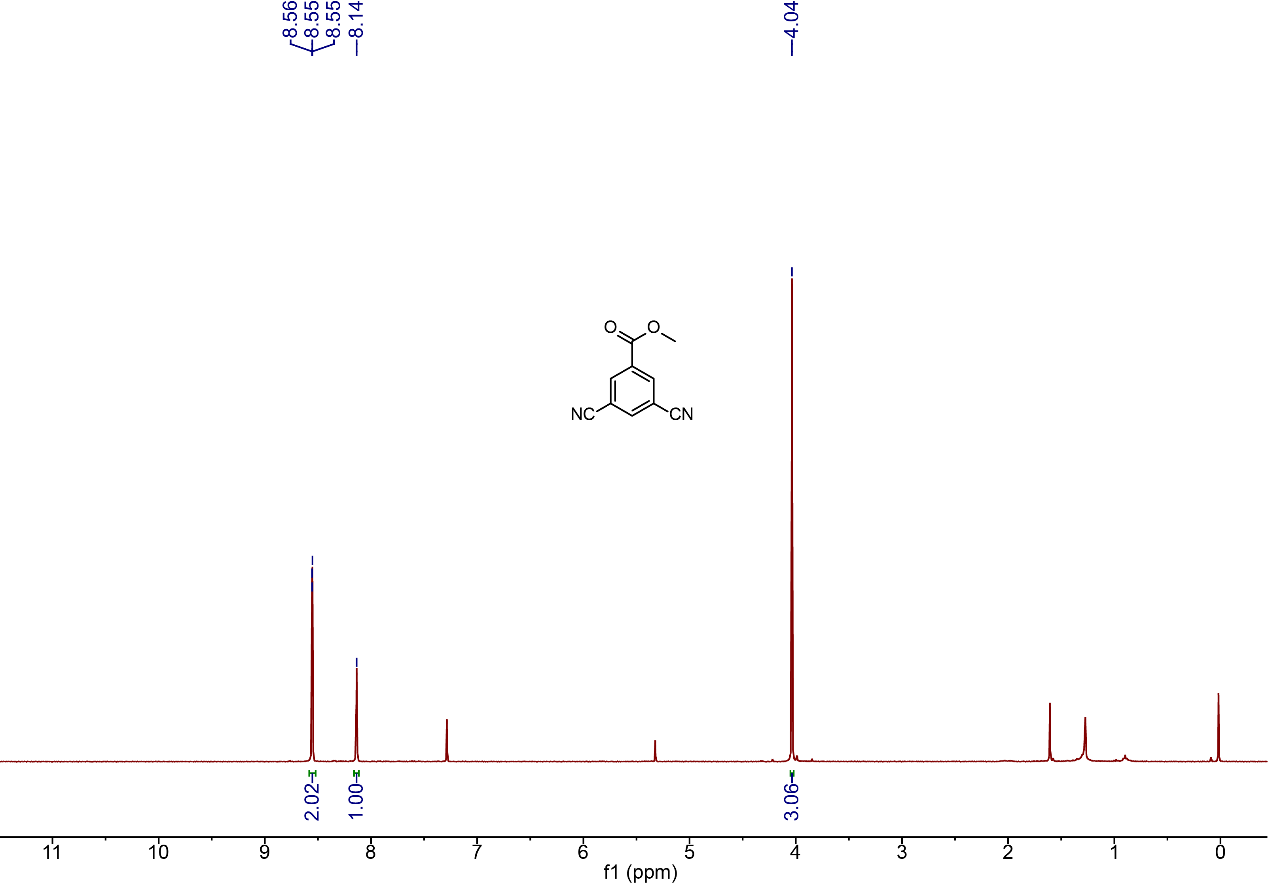
**

**Figure S3.** ^1^H NMR of **DCMe** in CDCl_3_.


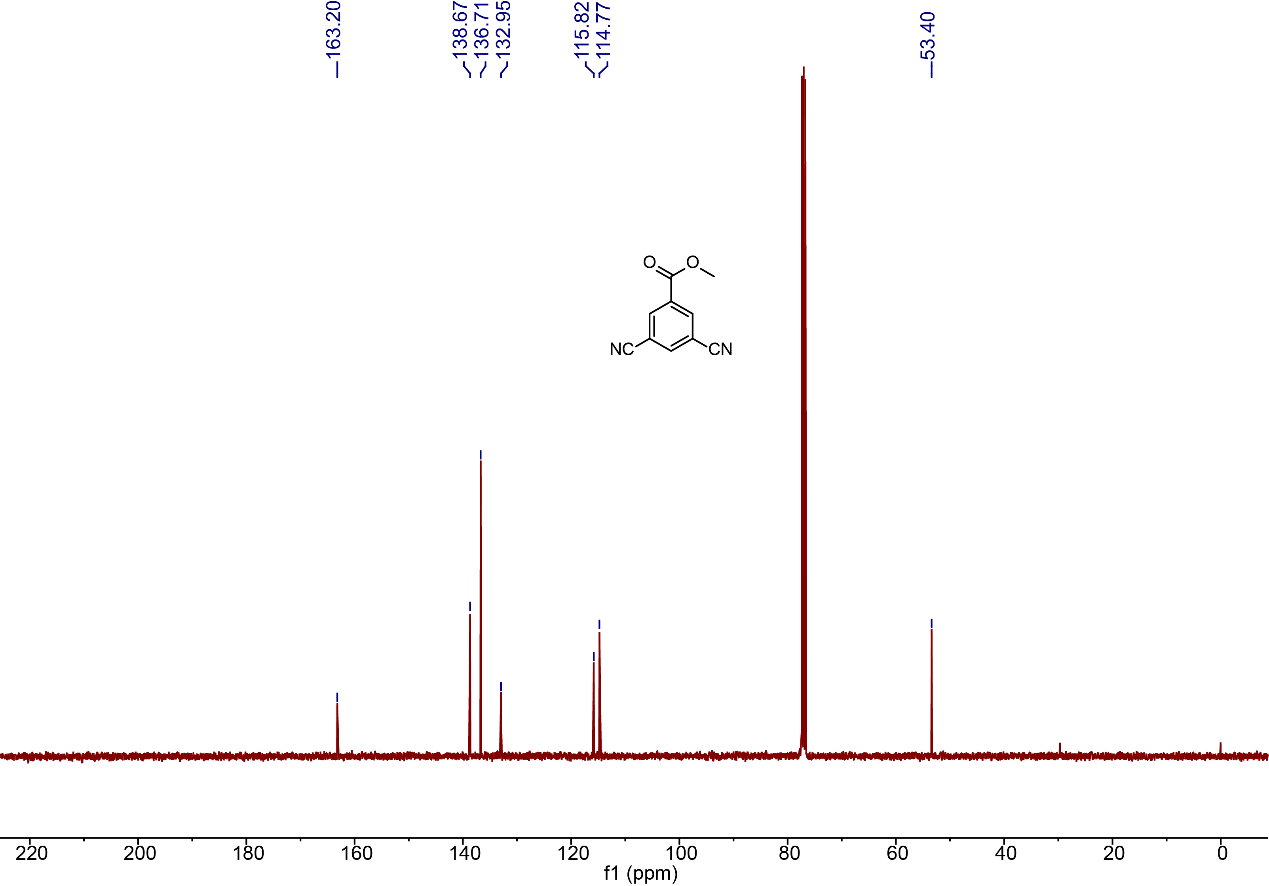


**Figure S4.** ^13^C NMR of **DCMe** in CDCl_3_.

**
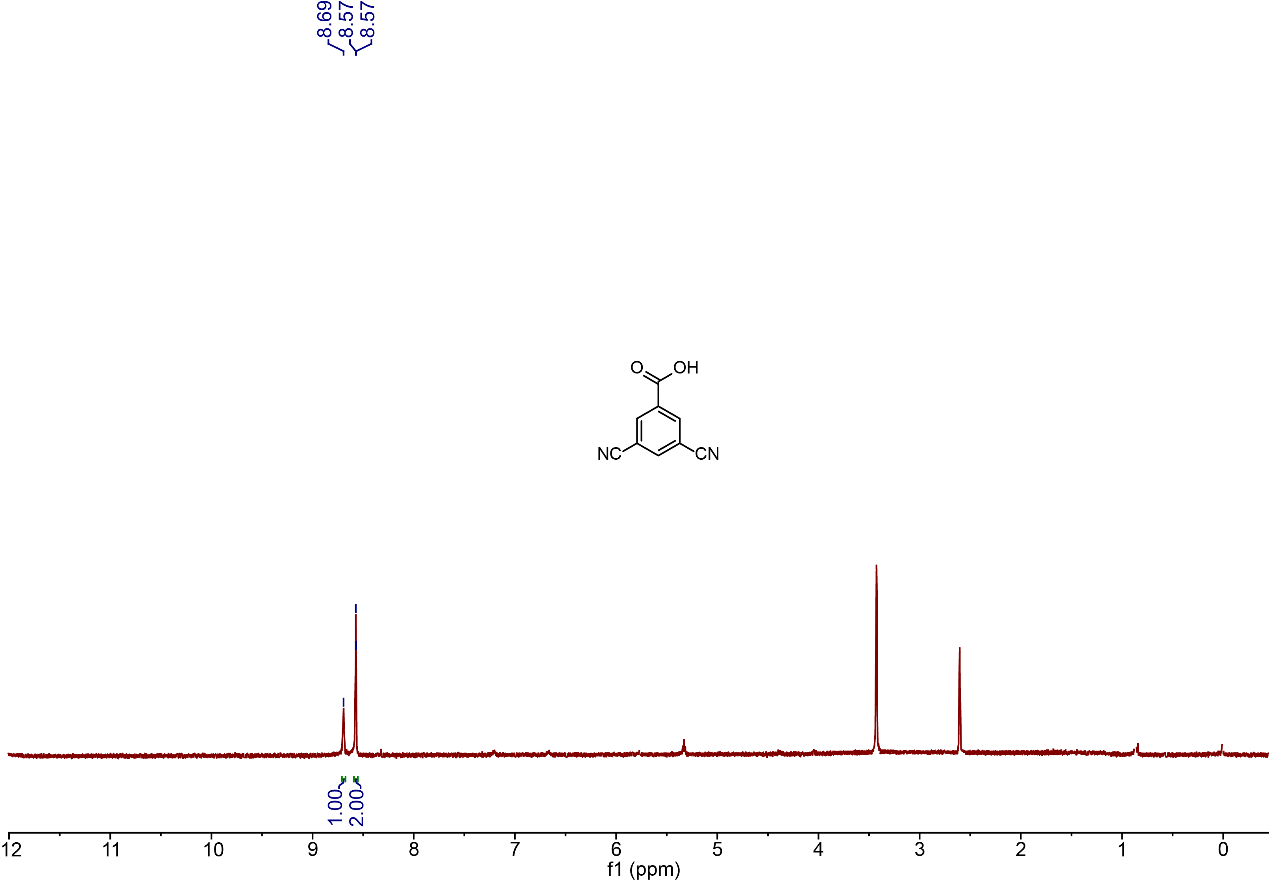
**

**Figure S5.** ^1^H NMR of **DCAc** in DMSO-d6.

**
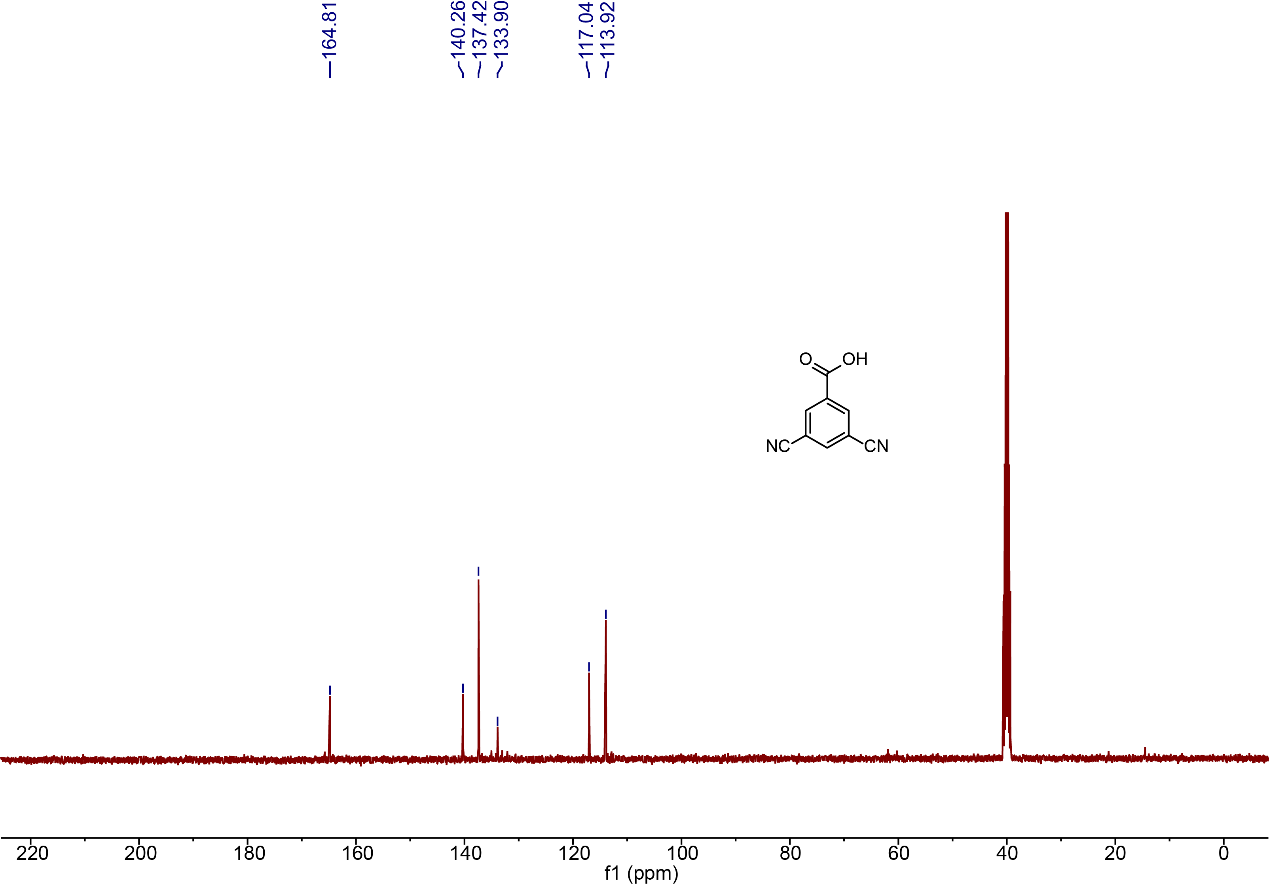
**

**Figure S6.** ^13^C NMR of **DCAc** in DMSO-d6.


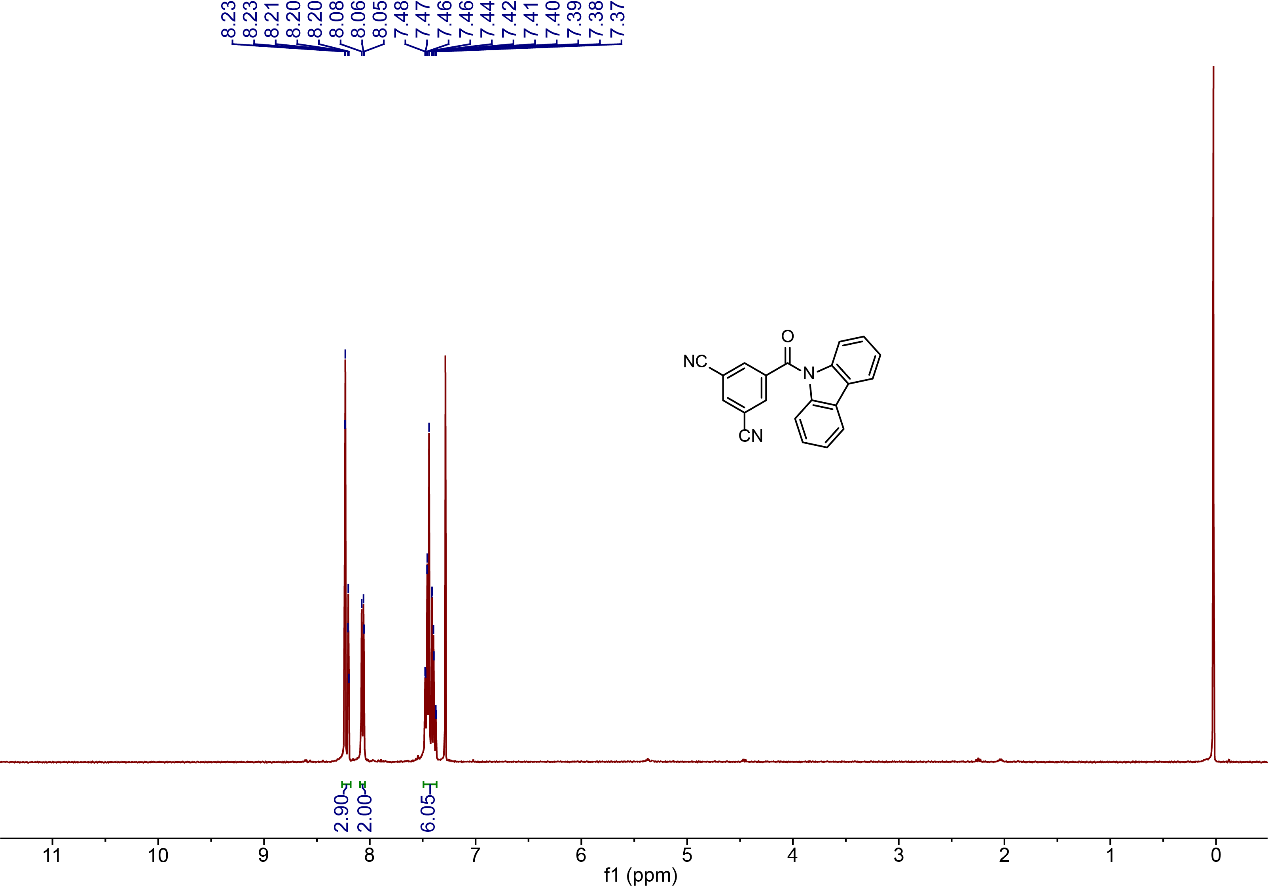


**Figure S7***.* ^1^H NMR of DCCz in CDCl_3_.

**
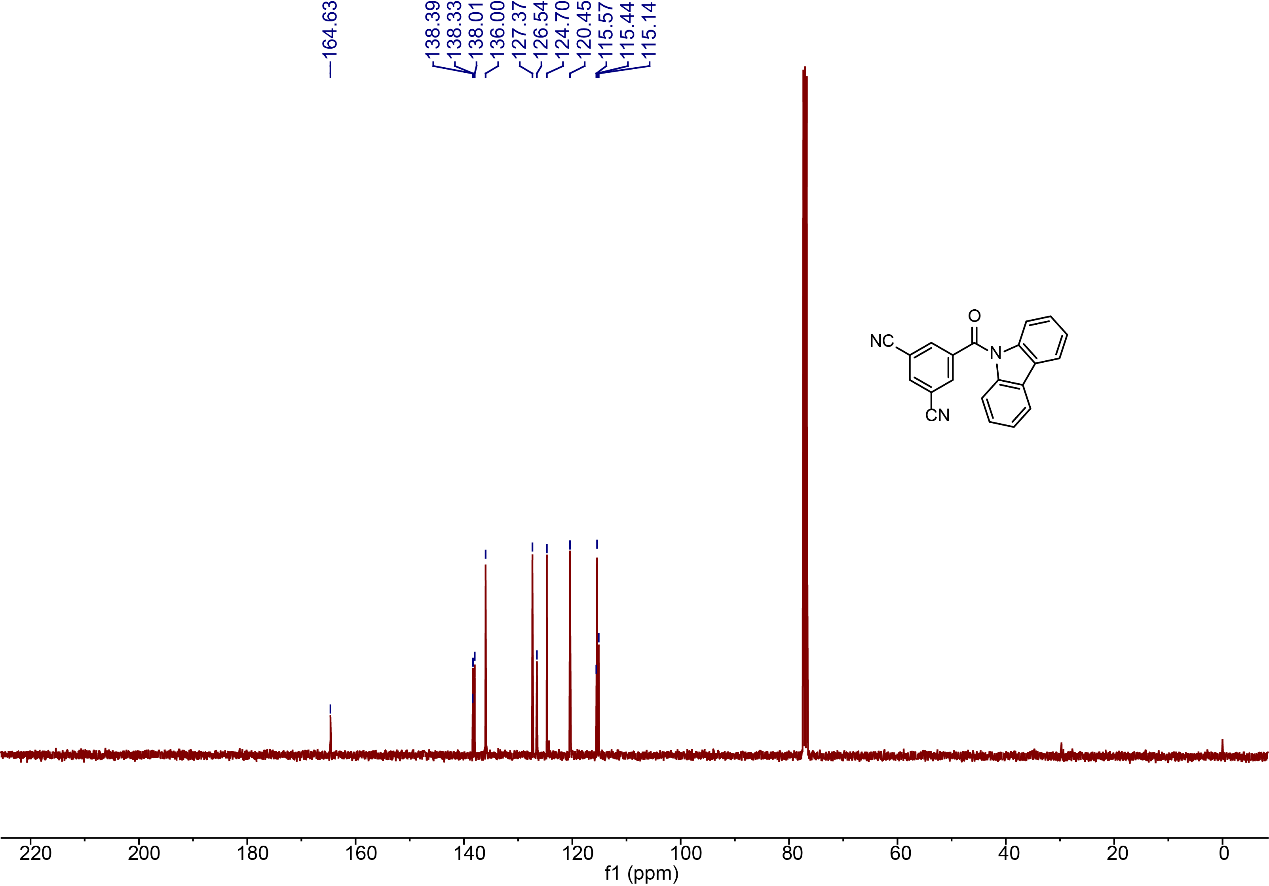
**

**Figure S8.** ^13^C NMR of DCCz in CDCl_3_.

# Thermal Property

Thermogravimetric analysis (TGA) was conducted on a Shimadzu DTG-60H thermogravimetric analysis under a heating rate of 10°C/min and a nitrogen flow rate of 50 cm^3^/min. The differential scanning calorimetry (DSC) analysis was performed on a Shimadzu DSC-60A instrument under a heating rate of 10°C/min and a nitrogen flow rate of 20 cm^3^/min. Thermal decomposition temperature (*T*_d_) is defined as the temperature when the sample’s weight loss reaches 5%.


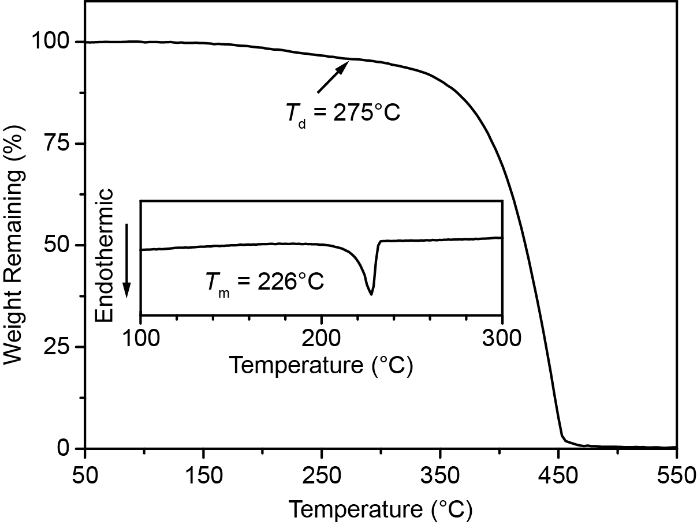


**Figure S9.** TGA and DSC curves of **DCCz**.

# Single Crystals Analysis

Colorless single crystal of **DCCz** was obtained by slow evaporation of a mixed DCM and petroleum ether (PE) solution at 295 K. The single crystal structure data was collected on a Bruker SMART APEX (II)-CCD at room temperature and crystal structures were analyzed by Mercury 4.0 software. Single crystal data were summarized in Table S1.

**Table S1.** Crystallographic data of **DCCz** at 295 K.

| Compound | **DCCz** |
| --- | --- |
| Formula | C_21_H_11_N_3_O |
| Formula weight (g mol^-1^) | 321.34 |
| Crystal color | colorless |
| Wavelength (Å) | 0.71073 |
| Crystal system | orthorhombic |
| Space group | p b c a |
| *a*, (Å) | 8.447(2) |
| *b*, (Å) | 16.957(4) |
| *c*, (Å) | 22.189(5) |
| *α*, (deg) | 90 |
| *β*, (deg) | 90 |
| *γ*, (deg) | 90 |
| volume, (Å^3^) | 3178.1(13) |
| *Z* | 8 |
| Density, (g cm^-3^) | 1.343 |
| *μ*, (mm^-1^) | 0.086 |
| F(000) | 1328 |
| *h*_max_, *k*_max_, *l*_max_ | 11, 18, 29 |
| *Theta*_max_ | 28.503 |
| CCDC number | 2036733 |

# Electrochemical Property

Cyclic voltammetry (CV) measurement was performed to estimate the highest occupied molecular orbital (HOMO) and the lowest unoccupied molecular orbital (LUMO) from the onset potential of the electrochemical oxidation and reduction waves, respectively.^[^[^2^](#_ENREF_2)^]^ The CV measurements were carried out at room temperature on a CHI660E system in a typical there-electrode cell with a working electrode (glass carbon), a reference electrode (Ag/Ag^+^), referenced against ferrocene/ferrocenium (FOC), and a counter electrode (Pt wire) in an acetonitrile (MeCN) solution of Bu_4_NPF_6_ (0.1 M) at a sweeping rate of 100 mV s^-1^. The thin solid film of the optoelectronic molecule was deposited on the surface of the glass carbon working electrode for CV measurement. HOMO and LUMO energy levels (*E*_HOMO_ and *E*_LUMO_) were estimated based on the reference energy level of ferrocene (4.8 eV below the vacuum) according to the following Equations:

$E_{HOMO}=-[E_{onset}^{Ox}-E_{(Fc/Fc^{+})}+4.8] eV$ (S1)

$E_{LUMO}=-[E_{onset}^{Red}-E_{(Fc/Fc^{+})}+4.8] eV$ (S2)

where *E*_(_*_Fc_*_/_*_Fc+_* _)_ is the onset oxidative voltage of FOC *vs* Ag/Ag^+^ and *EOx onset* and *ERed onset* are the onset potentials of the oxidation and reduction, respectively.


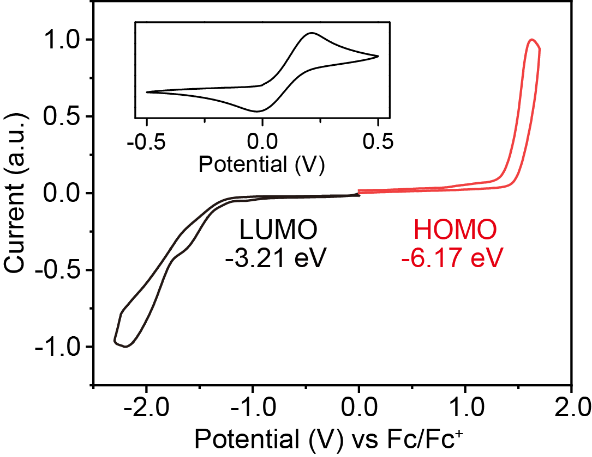


**Figure S10.** Cyclic voltammogram curve of **DCCz**. The insert graph shows the oxidation wave of ferrocene under the identical electrochemical conditions.

# Optical Properties

Ultraviolet-visible (UV-Vis) spectra were obtained using a SHIMADZU UV-3600 UV-VIS-NIR spectrophotometer. Steady-state and time-resolved photoluminescence were measured on an Edinburgh FLS980 fluorescence spectrophotometer. The absolute photoluminescence quantum yield (PLQY) was obtained using an Edinburgh FLS980 fluorescence spectrophotometer equipped with an integrating sphere. Phosphorescence spectra were obtained using an Edinburgh FLS980 fluorescence spectrophotometer with a 10 ms delay time after excitation using a microsecond flash lamp. The lifetimes (τ) of the luminescence were obtained by fitting the decay curve with a multi-exponential decay function of

where $A_{i}$ and $\tau_{i}$ represent the amplitudes and lifetimes of the individual components for multi-exponential decay profiles, respectively. X-ray activated RL spectra and X-ray photostability were obtained from an Edinburgh FS1000 fluorescence spectrophotometer equipped with a miniature X-ray source (AMPTEK, Inc.). Photo in the sunlight and X-ray imaging photographs were acquired with a digital camera (Canon, EOS R5 coupled with EF 100 mm f/2.8L IS USM) in an all-manual mode.

The photophysical properties of **DCCz** crystal presented in Table S3 (including the calculations of $k_{\mathrm{PF}}$, $k_{\mathrm{DF}}$, $k_{\mathrm{ISC}}$, and $k_{\mathrm{RISC}}$) and the corresponding derivation formulas (Eqs.S1-S6) are referenced to the “2.2. Key TADF Processes” section of the work by Tao et al.^[^[^3^](#_ENREF_3)^]^ Specifically, the calculations of prompt fluorescence efficiency ($\Phi_{\mathrm{PF}}$) and delayed fluorescence efficiency ($\Phi_{\mathrm{DF}}$) are based on the principle of distinguishing PF and DF components from total photoluminescence quantum yield via transient photoluminescence spectral integration (as described in the original section). Among them $\tau_{1}$=6.9 ns，$A_{1}$=7.69，$\tau_{2}$=984.4 ns，$A_{2}$=92.31，$\Phi_{total}$=32.1%.

$\Phi_{\mathrm{PF}}=\Phi_{\mathrm{total}}\frac{A_{1}}{A_{1}+A_{2}}$ (S3)

$\Phi_{\mathrm{DF}}=\Phi_{\mathrm{total}}\frac{A_{2}}{A_{1}+A_{2}}$ (S4)

$k_{\mathrm{PF}}=\frac{\Phi_{\mathrm{PF}}}{\tau_{\mathrm{PF}}}$ (S5)

$k_{\mathrm{DF}}=\frac{\Phi_{\mathrm{DF}}}{\tau_{\mathrm{DF}}}$ (S6)

$k_{\mathrm{ISC}}=\frac{\Phi_{\mathrm{DF}}}{\Phi_{\mathrm{DF}}+\Phi_{\mathrm{PF}}}k_{\mathrm{PF}}$ (S7)

$k_{\mathrm{RISC}}=\frac{k_{\mathrm{DF}}k_{\mathrm{PF}}}{k_{\mathrm{ISC}}}\frac{\Phi_{\mathrm{DF}}}{\Phi_{\mathrm{PF}}}$ (S8)


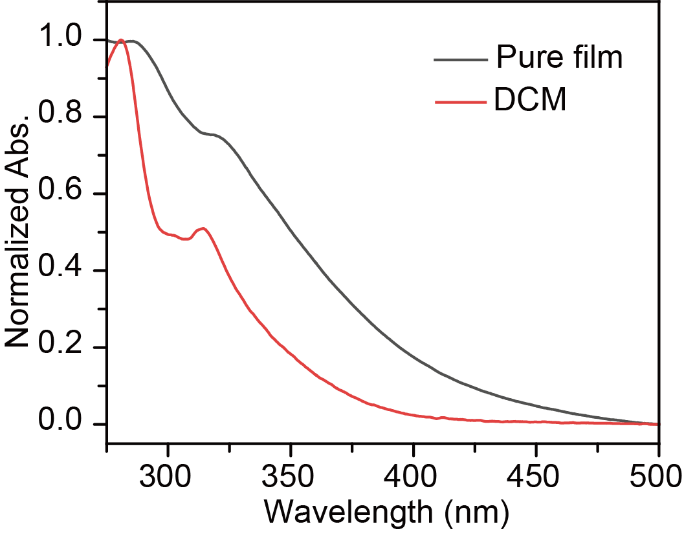


**Figure S11.** Absorption spectra of **DCCz** pure film and in DCM solutions with concentrations of 10^−5^ M under ambient conditions.


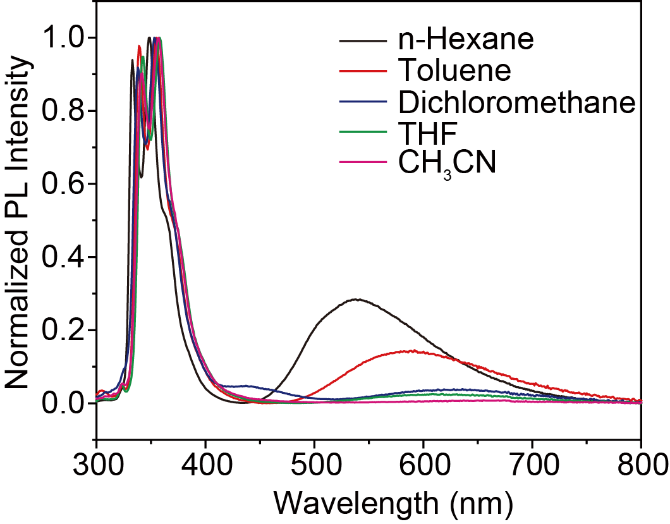


**Figure S12.** PL spectra of **DCCz** in n-hexane, toluene, dichloromethane (DCM), tetrahydrofuran (THF) and acetonitrile (MeCN) solutions with concentrations of 10^−5^ M under ambient conditions.


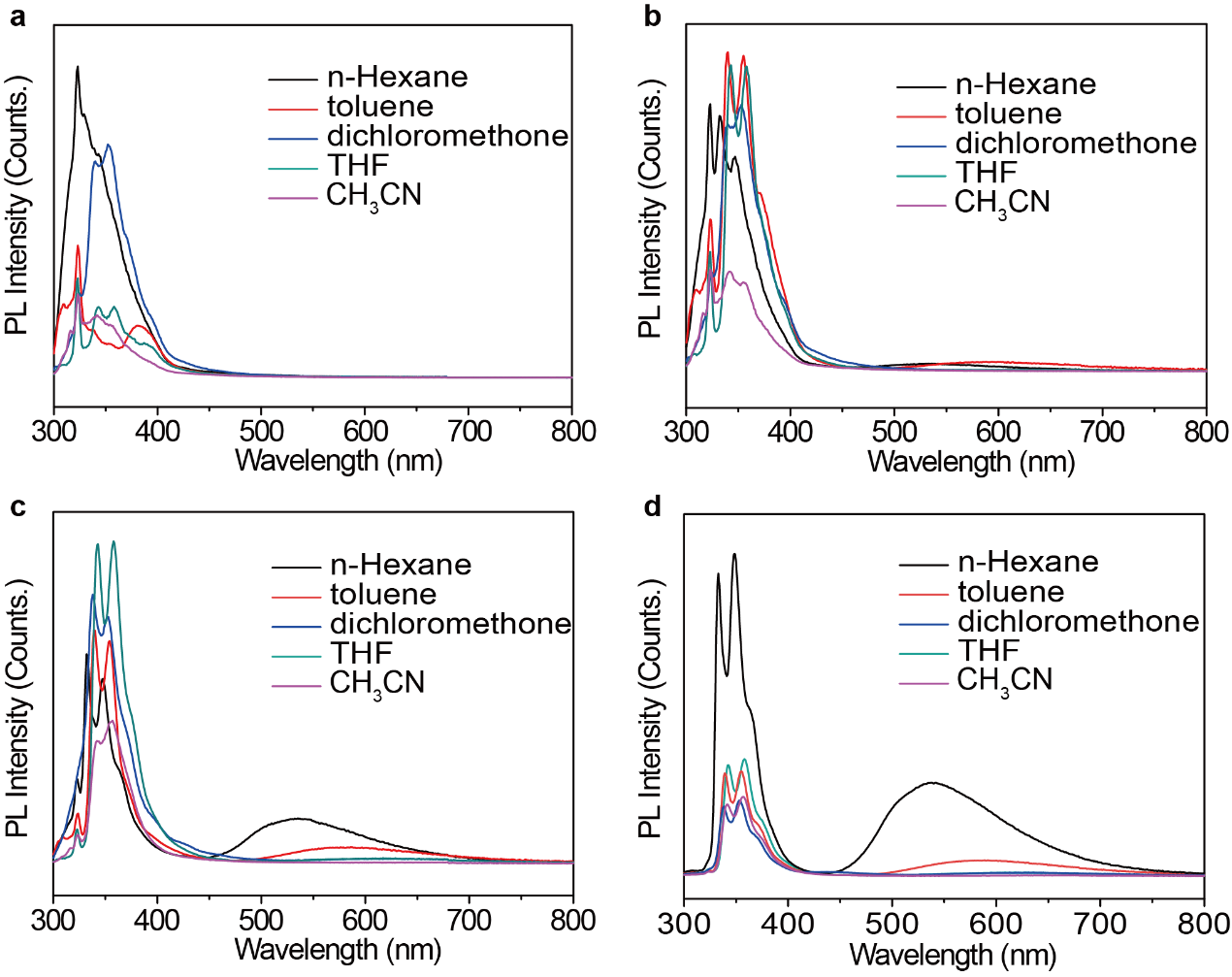


**Figure 13.** Steady-state photoluminescence spectra of compound **DCCz** under UV excitation (295 nm) in different solvents at concentrations of (a) 10⁻^7^ M, (b) 10⁻^6^ M, (c) 10⁻^5^ M, and (d) 10⁻^4^ M.


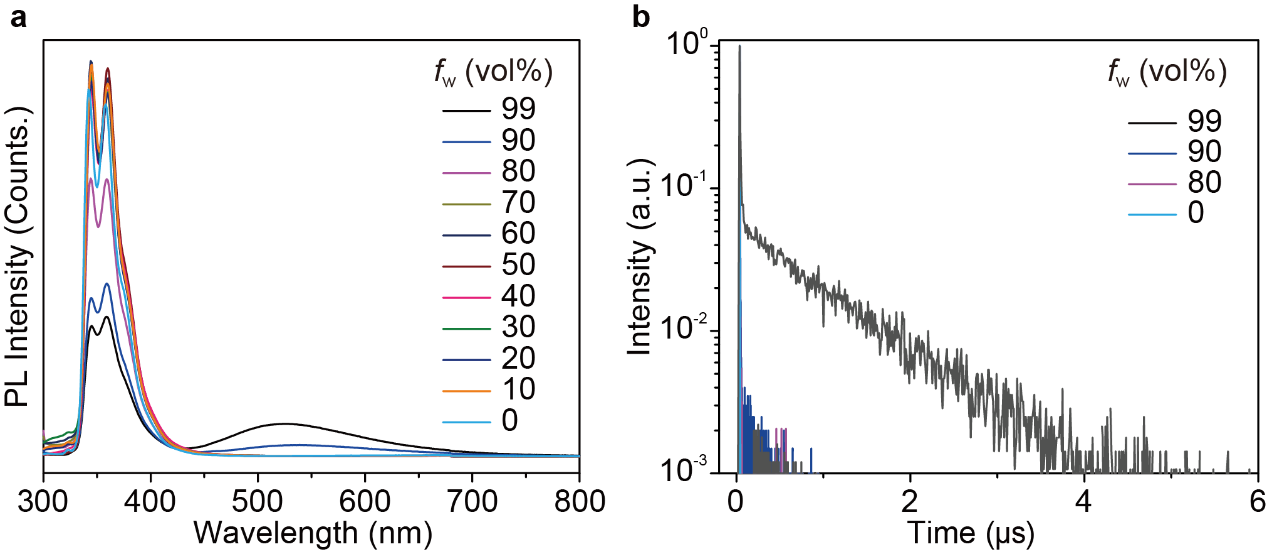


**Figure S14.** PL spectra (a) and lifetime decay (b) profiles of **DCCz** in THF/water mixtures with different water fractions (f_w_).

**Table S2.** Fitting parameters for the Temperature-dependent transient PL decay curves (80k-300k) of **DCCz** in crystal.

| Temperature | $\boldsymbol{A}_{\boldsymbol{1}}$ | $\boldsymbol{\tau}_{\boldsymbol{1}}$ | $\boldsymbol{A}_{\boldsymbol{2}}$ | $\boldsymbol{\tau}_{\boldsymbol{2}}$ |
| --- | --- | --- | --- | --- |
| 80 K | 42.52% | 4.27 ns | 57.48% | 3895.66 ns |
| 120 K | 27.00% | 4.52 ns | 73.00% | 3720.28 ns |
| 160 K | 18.75% | 4.15 ns | 81.25% | 2845.49 ns |
| 200 K | 15.61% | 4.32 ns | 84.39% | 2026.95 ns |
| 240 K | 12.08% | 4.22 ns | 87.92% | 1698.85 ns |
| 280 K | 11.08% | 4.17 ns | 88.92% | 1117.48 ns |
| 300 K | 8.53% | 4.28 ns | 91.47% | 838.09 ns |

**Table S3.** Photophysical properties of **DCCZ** crystal.

| $\boldsymbol{k}_{\mathbf{PF}}$ | $\boldsymbol{k}_{\mathbf{DF}}$ | $\boldsymbol{k}_{\mathbf{ISC}}$ | $\boldsymbol{k}_{\mathbf{RISC}}$ | $\boldsymbol{\Phi}_{\mathbf{PF}}$ | $\boldsymbol{\Phi}_{\mathbf{DF}}$ |
| --- | --- | --- | --- | --- | --- |
| 3.58×10^6^ s^−1^ | 3.01×10^5^ s^−1^ | 3.31×10^6^ s^−1^ | 3.91×10^6^ s^−1^ | 2.47 % | 29.63 % |


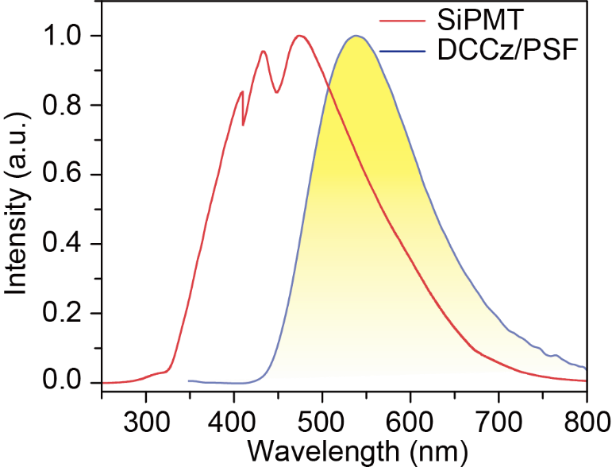


**Figure S15.** Spectral response range of SiPM and RL spectrum of **DCCz**/PSF film.

# Theoretical Calculations

The geometries of the studied molecular ground states were optimized using density-functional theory (DFT) and computational analyses were performed on the same level to determine that the optimized geometries are stable configurations. The universal B3LYP hybridization generalization and the 6-31G(d) basis group were used, combined with DFT-D3(BJ) dispersion corrections because of the weak interactions involved. All theoretical calculations were performed under the Gaussian09D.01 program package, and images of the front molecular orbitals were obtained by visual molecular dynamics (VMD).^[^[^4^](#_ENREF_4)^]^ The exchange integrals were analyzed using the Multiwfn 3.8 program package.^[^[^5^](#_ENREF_6)^]^

$E_{S}=E+K+J$ $E_{T}=E+K-J$ ${\Delta E}_{ST}=E_{S}-E_{T}=2J$ (S9)

where *E* is orbital energy, *K* is repulsion energy and *J* is exchange energy.

$J=\iint\varphi_{L}(1)\varphi_{H}(1)(\frac{e^{2}}{r_{1}-r_{2}})\varphi_{L}(2)\varphi_{H}(2)dr_{1}dr_{2}$ (S10)

The results are as follows:

Monomer: *J*=0.12 eV Dimer: *J*=0.006 eV

**Table S4.** Electronic energy levels of the **DCCz**, calculated (dimer) *vs* experimental (in doped film). *​*

|  | Calculated | Experimental |
| --- | --- | --- |
| HOMO | -6.14 eV | -6.17 eV |
| LUMO | -2.49 eV | -3.21 eV |
| Δ*E*_ST_ | 0.01 eV | 0.01 eV |

# Fabrication of Scintillation Screen

The detailed preparation processes of **DCCz**@PSF film were showed as follows: Firstly, polysulfone (PSF) and the **DCCz** are placed in a beaker, dissolved in chloroform, and stirred thoroughly to form a homogeneous mixture. Subsequently, the stirred mixture solution is poured onto a clean glass plate. After that, the mixture on the glass plate undergoes an evaporation process for 24 hours, allowing the solvent in the mixture to gradually volatilize. Finally, an X-ray screen is successfully prepared. The photoelectric sensor employed in this study is a Canon EOS R5. For static imaging measurements, the parameters were set as follows: Aperture: f/2.8; Exposure time: 20 s; ISO sensitivity: 6400; Focal length: 100 mm; Imaging distance: 0.6 m. The value of 19.7 lp·mm-1 refers to the spatial frequency at which the modulation transfer function (MTF) reaches 0.2, obtained via edge spread function analysis (Figure 4b). By contrast, 20 lp·mm-1 is the experimentally determined resolution limit using a standard line-pair test pattern under real imaging conditions.


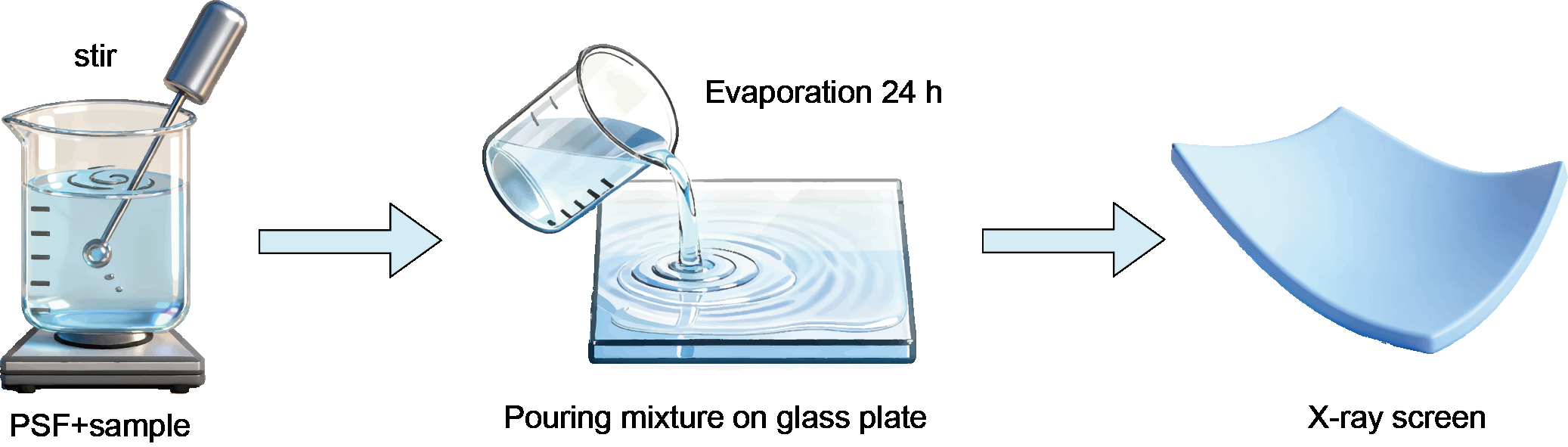


**Figure S16.** Schematic diagram illustrating the preparation procedure of the scintillator film.


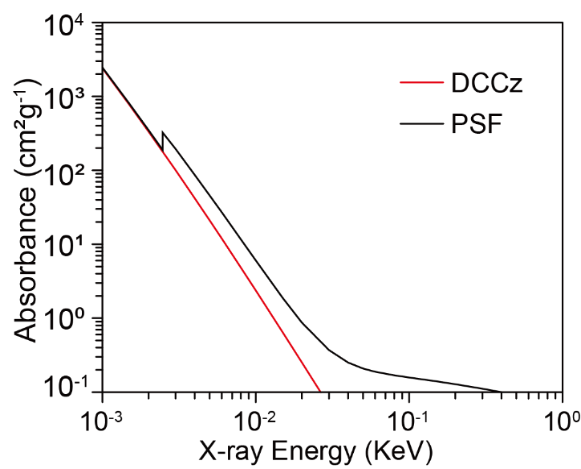


**Figure S17.** X-ray absorption spectrum of **DCCz** and PSF.


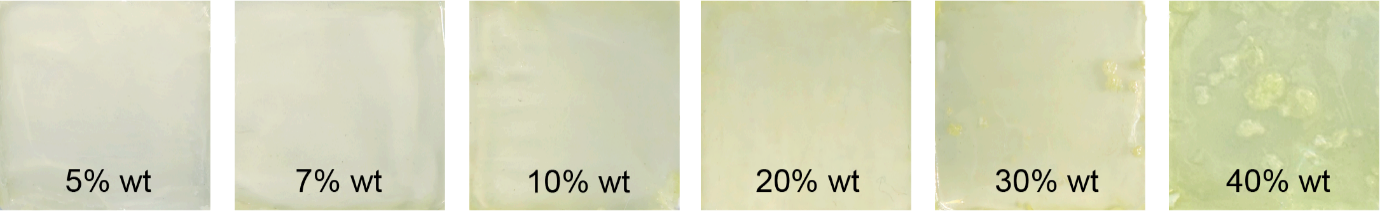


**Figure S18.** Photographs of **DCCz**/PSF films with different doping concentrations (5 wt%, 7 wt%, 100 wt%, 20 wt%, 30 wt%,and 40 wt%) under daylight.

**Table S5**. Comparison for parameters of scintillators for lifetime and X-ray imaging.

| Scintillator | Emission  Peak (nm) | Lifetime | Spatial  Resolution (lp mm^-1^) | Ref | | |
| --- | --- | --- | --- | --- | --- | --- |
| 1FAT | 523 | 1.514 μs | 20.6@MTF=0.2 | [[6](#_ENREF_7)] | | |
| AIDF-Br | 533 | 478.4 ns | [29.2@MTF=0.2](mailto:29.2@MTF=0.2) | [[7](#_ENREF_8)] | | |
| TPE-4Br | 448 | 1.79 ns | 18.69@MTF=0.2 | [[8](#_ENREF_9)] | | |
| TADF-Br | 505 | 1.42 μs | 12@MTF=0.2 | [[9](#_ENREF_10)] | | |
| DPXZ-3-TpBO | 515 | 20.4 μs | 13@MTF=0.2 | [[10](#_ENREF_11)] | | |
| DMAc-TRZ | 500 | 2.35 μs | 16.6@MTF=0.2 | [[11](#_ENREF_12)] | | |
| (MTP)_3_(Eu_9_H_2_OCl_6_•5.75H_2_O | 592 | 118.48 μs | 13.23@MTF=0.2 | [[12](#_ENREF_13)] | | |
| [LB]₂Cu₄I₆ | 541 | 2.93 μs | 10.3@MTF=0.2 | [[13](#_ENREF_14)] | | |
| **DCCz** | 538 | 973.9 ns | 19.7@MTF=0.2 | | This work |  |

**References**

[1] D. H. Ahn, S. W. Kim, H. Lee, I. J. Ko, D. Karthik, J. Y. Lee, J. H. Kwon, *Nat. Photonics.* **2019**, 13, 540.

[2] H. Li, R. Bi, T. Chen, K. Yuan, R. Chen, Y. Tao, H. Zhang, C. Zheng, W. Huang, *ACS Appl. Mater. Interfaces* **2016**, 8, 7274.

[3] Y. Tao, K. Yuan, T. Chen, P. Xu, H. Li, R. Chen, C. Zheng, L. Zhang, W. Huang, *Adv. Mater.* **2014**, 26, 7931.

[4] a)M. J. Frisch, G. W. Trucks, H. B. Schlegel, G. E. Scuseria, M. A. Robb, J. R. Cheeseman, G. Scalmani, V. Barone, B. Mennucci, G. A. Petersson, H. Nakatsuji, M. Caricato, X. Li, H. P. Hratchian, A. F. Izmaylov, J. Bloino, G. Zheng, J. L. Sonnenberg, M. Hada, M. Ehara, K. Toyota, R. Fukuda, J. Hasegawa, M. Ishida, T. Nakajima, Y. Honda, O. Kitao, H. Nakai, T. Vreven, J. A. Montgomery, J. E. Peralta, F. Ogliaro, M. Bearpark, J. J. Heyd, E. Brothers, K. N. Kudin, V. N. Staroverov, R. Kobayashi, J. Normand, K. Raghavachari, A. Rendell, J. C. Burant, S. S. Iyengar, J. Tomasi, M. Cossi, N. Rega, J. M. Millam, M. Klene, J. E. Knox, J. B. Cross, V. Bakken, C. Adamo, J. Jaramillo, R. Gomperts, R. E. Stratmann, O. Yazyev, A. J. Austin, R. Cammi, C. Pomelli, J. W. Ochterski, R. L. Martin, K. Morokuma, V. G. Zakrzewski, G. A. Voth, P. Salvador, J. J. Dannenberg, S. Dapprich, A. D. Daniels, Farkas, J. B. Foresman, J. V. Ortiz, J. Cioslowski, D. J. Fox, Gaussian 09, rev. D.01; Gaussian, Inc.: Wallingford, CT, **2009**; b) Humphrey, W.; Dalke, A.; Schulten, K. Vmd: Visual Molecular Dynamics*. J. Mol. Graphics.* **1996**, 14, 33.

[5] T. Lu, F. Chen, *J. Comput. Chem..* **2011**, 33, 580.

[6] J. Sun, M. Ding, H. Ma, X. Wang, M. Li, H. Wang, J. Du, Z. Zhou, A. Lv, H. Wang, Z. An, H. Shi, W. Huang, *Adv. Mater.* **2025**, 37,2507058.

[7] H. Wang, Q. Sun, F. Yang, Z. Li, L. Sun, X. Zhang, Z. Zhao, W. Hu, *SmartMat.* **2025**, 6, e70002.

[8] X. Du, S. Zhao, L. Wang, H. Wu, F. Ye, K.-H. Xue, S. Peng, J. Xia, Z. Sang, D. Zhang, Z. Xiong, Z. Zheng, L. Xu, G. Niu, J. Tang, *Nat. Photonics.* **2024**, 18, 162.

[9] J.-X. Wang, L. Gutiérrez-Arzaluz, X. Wang, T. He, Y. Zhang, M. Eddaoudi, O. M. Bakr, O. F. Mohammed, *Nat. Photonics.* **2022**, 16, 869.

[10] W. Yang, C. Xie, T. Chen, X. Yin, Q. Lin, S. Gong, Z. Quan, C. Yang, *Angew. Chem. Int. Ed.* **2024**, 63, e202402704.

[11] W. Ma, Y. Su, Q. Zhang, C. Deng, L. Pasquali, W. Zhu, Y. Tian, P. Ran, Z. Chen, G. Yang, G. Liang, T. Liu, H. Zhu, P. Huang, H. Zhong, K. Wang, S. Peng, J. Xia, H. Liu, X. Liu, Y. M. Yang, *Nat. Mater.* **2021**, 21, 210.

[12] C. Zhao, Y. Wang, S. Bao, Y. Zang, X. Liu, W. Huang, *Adv. Mater.* **2025**, 37, 2500925.

[13] L. Lian, D. Xiong, J. Zhang, M. Jia, Y. Liu, Z. Ma, X. Chen, Y. Han, Y. Tian, X. Li, J. Zhang, S. Qian, C. Shan, Z. Shi, *Appl. Phys. Rev.* **2025,** 12, 031409.
